# Lipid metabolic reprogramming in tumor microenvironment: from mechanisms to therapeutics

**DOI:** 10.1186/s13045-023-01498-2

**Published:** 2023-09-12

**Authors:** Hao-Ran Jin, Jin Wang, Zi-Jing Wang, Ming-Jia Xi, Bi-Han Xia, Kai Deng, Jin-Lin Yang

**Affiliations:** 1 Department of Gastroenterology and Hepatology, West China Hospital, Sichuan University, No.37 Guoxue Road, Wuhou District, Chengdu, 610041 China; 2 Sichuan University-University of Oxford Huaxi Joint Centre for Gastrointestinal Cancer, Frontiers Science Center for Disease-Related Molecular Network, West China Hospital, Sichuan University, Chengdu, China

**Keywords:** Lipid metabolism, Tumor microenvironment, Cancer progression, Immune response, Targeted therapy

## Abstract

Lipid metabolic reprogramming is an emerging hallmark of cancer. In order to sustain uncontrolled proliferation and survive in unfavorable environments that lack oxygen and nutrients, tumor cells undergo metabolic transformations to exploit various ways of acquiring lipid and increasing lipid oxidation. In addition, stromal cells and immune cells in the tumor microenvironment also undergo lipid metabolic reprogramming, which further affects tumor functional phenotypes and immune responses. Given that lipid metabolism plays a critical role in supporting cancer progression and remodeling the tumor microenvironment, targeting the lipid metabolism pathway could provide a novel approach to cancer treatment. This review seeks to: (1) clarify the overall landscape and mechanisms of lipid metabolic reprogramming in cancer, (2) summarize the lipid metabolic landscapes within stromal cells and immune cells in the tumor microenvironment, and clarify their roles in tumor progression, and (3) summarize potential therapeutic targets for lipid metabolism, and highlight the potential for combining such approaches with other anti-tumor therapies to provide new therapeutic opportunities for cancer patients.

## Introduction

Metabolic reprogramming has emerged as a critical feature of cancer. To adapt to the hypoxic and nutrient-poor microenvironment, in addition to increasing glucose uptake and aerobic glycolysis, tumor cells also undergo lipid metabolism reprogramming to enhance their biological behaviors [1]. This is characterized by increased lipid uptake, lipid synthesis, fatty acid oxidation (FAO), and lipid storage. Mounting evidence demonstrated that lipids play a critical role in cancer progression by serving as energy sources, membrane structures, signaling molecules (including bioactive lipids like S1P, PGE2, and LPA), and even causing epigenetic modifications through fatty acylation of key molecules [2, 3]. Mechanically, alterations in lipid metabolic phenotype in tumor cells are directly driven by continuous oncogenic events and extracellular tumor microenvironment (TME) factors such as hypoxia, acidosis, and nutritional alterations [4, 5].

In addition to supporting tumor development, lipid metabolic reprogramming also modifies the TME by influencing the recruitment, activation, and function of immune cells and stromal cells. Tumor cells and cells in TME interact with each other and form a reciprocal entity [6]. On one hand, tumor cells can actively modify the TME by secreting signaling molecules and metabolites, which affect the functions of cancer-associated fibroblasts (CAFs) and immune cells in TME [6]. On the other hand, lipid metabolic reprogramming, an adaptive change in cells within the TME, manifests as increased lipid uptake and accumulation, or FAO, driving the TME toward an immunosuppressive phenotype supporting tumor progression [7]. For example, upregulated lipid uptake and FAO increase lipid metabolic levels in regulatory T cells (Tregs), tumor-associated macrophages (TAMs), and myeloid-derived suppressor cells (MDSCs), facilitating their immunosuppressive function [8–10]. Moreover, upregulation of CD36 in CD8^+^ T cells leads to excessive lipid accumulation, which impairs secretion of anti-tumor factors such as IFN-γ and TNF-α, ultimately suppressing their anti-tumor efficacy [11, 12]. Similarly, upregulation of CD36 in natural killer (NK) cells also impairs their tumor-killing activity through intracellular lipid accumulation. Studies have suggested that blocking lipid uptake via inhibition of CD36 on cytotoxic CD8^+^ T cells or Tregs enhances anti-tumor immune responses [8, 11].

Given the critical role of lipids in cancer progression, targeting to lipid metabolism-related pathways offers new therapeutic opportunities for cancer. A large body of evidence shows that inhibitors targeting lipid uptake, lipogenesis, and FAO in tumor cells have shown significant therapeutic effects in various cancers [13–15]. Besides, modulating lipid metabolism in stromal cells and immune cells also provides a new choice for anti-tumor therapy. Moreover, it can be combined with chemotherapy and immunotherapy, providing a new comprehensive strategy for optimizing cancer treatment. This review aims to clarify the lipid metabolic landscape in tumor cells and TME cells, and summarize potential targets to offer clues for further research and clinical applications of targeting lipid metabolism in cancer.

## Landscape and mechanisms of lipid metabolic reprogramming in cancer

### Lipid metabolic reprogramming in cancer

**Fig. 1:**
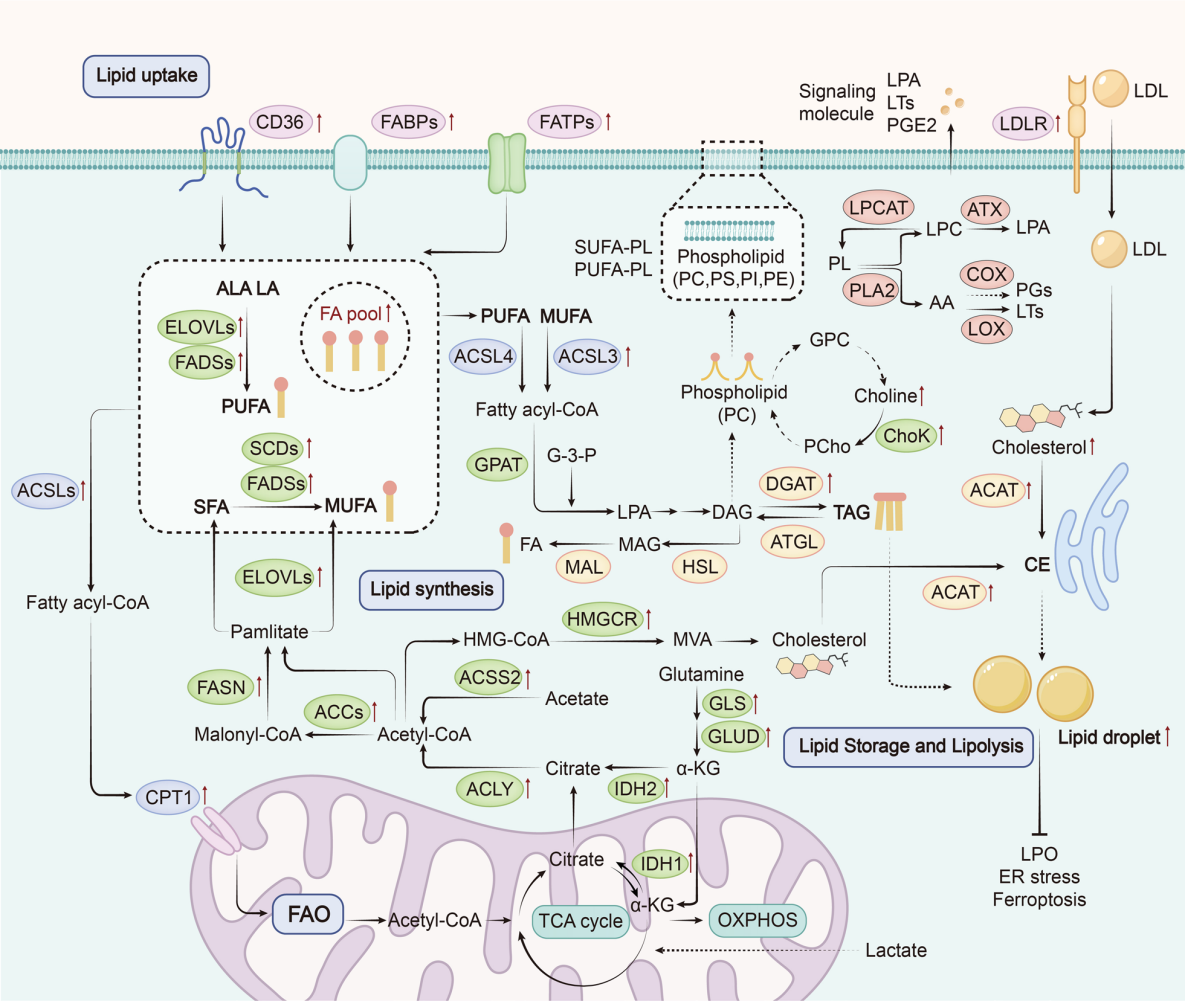
Lipid metabolic reprogramming in cancer. Tumor cells enhance lipid metabolism by increasing exogenous lipid uptake and lipid synthesis, leading to increased intracellular lipid content. Upregulation of lipid transport proteins such as CD36, FATPs, FABPs, and LDLR increases lipid uptake. These upregulations increase intracellular SUFA, PUFA, and cholesterol levels. Meanwhile, endogenous lipid synthesis originates from citrate in the TCA cycle, as well as intracellular glutamine, lactate and acetate, leading to the synthesis of SFA and cholesterol. The process is catalyzed by key enzymes such as FASN and SCD. FAs in tumor cells are catalyzed by ACSL to form acyl-CoA, which is involved in the subsequent synthesis of intracellular phospholipids with bioactive lipids and FAO. Acyl-CoA facilitates the translocation of enzymes into mitochondria through CPT1, the key enzyme of FAO, participating in the production of acetyl-CoA, providing energy for the biological behavior of tumor cells. Excess lipids in tumor cells are stored in LD as CE and TAG. This storage significantly prevents LPO and attenuates its risk of mediating tumor cell death

Most lipid molecules in the human diet are triacylglycerols (TAGs) and cholesterol. After absorption, TAGs can be hydrolyzed into glycerol and fatty acids (FAs). Glycerol is then converted into glycerol-3-phosphate (G-3-P), which enters glycolysis. FAs can either be stored as the primary component of membrane synthesis or converted to acyl-CoA for β-oxidation to provide energy. In tumors, several steps of lipid metabolism show universal enhancement to maintain their biological progressions. This includes increased lipid uptake, synthesis, storage, and FAO. To delve deeper into these lipid metabolic alterations, the following section provides an in-depth analysis (Fig. 1).

#### Lipid uptake in cancer

The increase in intracellular lipid content can be achieved through two pathways: endogenous and exogenous pathways. Endogenous lipids are primarily produced through de novo lipogenesis (DNL), which utilizes acetyl-CoA as a substrate. Exogenous lipids require the involvement of transport molecules, including CD36, fatty acid transport protein family (FATPs/SLC27), and fatty acid-binding proteins (FABPs) [4]. Notably, recent studies have established a link between the overexpression of these transport molecules and the poor prognosis across various cancers. For instance, CD36 overexpression is associated with a poor prognosis in breast, ovarian, gastric, colorectal, and prostate cancer [15]. Moreover, FABPs have been found to contribute to the promotion of cervical cancer metastasis by increasing intracellular Fas [16], while knockdown of FABPs suppressed tumor progression in vivo by inhibiting lipid uptake in glioblastoma [17]. FATP members have been implicated in cancer initiation and progression in melanoma and breast cancer in multiple studies [18, 19].

#### Lipid synthesis in cancer

Although the exogenous lipid sources increase, cancer cells also activate DNL to respond to their high metabolic demands[2]. This pathway begins with acetyl-CoA, which can be mainly generated from citrate, a substrate in the TCA cycle during nutrient catabolism, via ATP-citrate lyase (ACLY). Besides, acetate conversion via acetyl-CoA synthetase (ACSS) is another pathway to produce acetyl-CoA for DNL. Acetyl-CoA is activated by acetyl-CoA carboxylases (ACCs) to form malonyl-CoA, which is further catalyzed by fatty acid synthase (FASN) to form saturated fatty acids (SFA), palmitate (C16:0). The resulting palmitate can be elongated by elongation of very-long-chain fatty acids gene family (ELOVLs) and desaturated by stearoyl-CoA desaturases (SCDs) or fatty acid desaturases (FADSs) to synthesize monounsaturated fatty acid (MUFA), such as oleic acid (OA) (C18:1) and palmitoleic acid (C16:1). Moreover, desaturation caused by ELOVLs and FADSs converts ingested polyunsaturated fatty acids (PUFAs) like linoleic acid (LA) (C18:2) and alpha-linolenic acid (ALA) (C18:3) into other PUFAs like arachidonic acid (AA) (C20:4) and adrenic acid (AdA) (C22:4) [20, 21]. Interestingly, overexpression or increased activity of ACLY has been correlated with the progression of various cancers [22]. ACSS is transcriptionally upregulated by SREBP, highly expressed in tumor cells, and plays a role in maintaining cancer cell growth under nutrient deficiency by catalyzing acetate [23]. FASN is commonly overexpressed in many epithelial and precancerous lesions and is associated with a high risk of cancer recurrence and mortality [24]. Inhibition of FASN can suppress breast cancer growth in the brain, highlighting its potential as a therapeutic target for metastasis in breast cancer [25]. SCD1 facilitates the formation of MUFAs, including OA, and its increased expression has been shown to promote the progression of cancers [26, 27].

In oncogenic processes, tumor cells utilize other metabolic substances in the microenvironment, such as glutamine and lactate, as sources of lipid synthesis. Glutamine dependence has been considered a metabolic hallmark of cancer cells. A growing body of evidence has shown that glutamine uptake and synthesis is upregulated in various cancers [28]. Cellular glutamine undergoes a transformation into α-ketoglutarate through the activation of glutaminase (GLS) and glutamate dehydrogenase (GLUD), ensuring the replenishment of vital metabolic intermediates within the TCA cycle. Subsequently, α- ketoglutarate is carboxylated by isocitrate dehydrogenase (IDH) to generate citrate [29]. In addition to glutamine, lactate is also an important source of TCA cycle intermediates and acetyl-CoA [30]. A recent study identified that lactate in the TME can reprogram lipid metabolism by increasing the expression of the genes involved, promoting tumor progression [31]. Notably, lactate promotes glutamine uptake and catabolism in oxidative cancer cells [32]. Therefore, utilizing glutamine and lactate to produce acetyl-CoA as a source of lipid synthesis is one of the important indirect ways for tumor cells to regulate lipid metabolism.

The synthesis of triacylglycerol (TAG) from long-chain fatty acids (LCFAs) derived from lipid intake and DNL involves a series of enzymatic reactions. Specifically, glycerol-3-phosphate acyltransferase (GPAT) catalyzes the combination of LCFAs with G-3-P to generate lysophosphatidic acid (LPA), which is a crucial intermediate in TAG synthesis. LPA is then converted to diacylglycerol (DAG) and subsequently to TAG via diacylglycerol acyltransferase (DGAT) [33]. Notably, DAG is also involved in compound lipid synthesis, such as cholesterol and phospholipids, which play critical roles in supporting key oncogenic functions and cancer hallmarks, and in regulating intercellular communication and immune responses [34].

Cholesterol, like other lipids, relies on acetyl-CoA for intracellular synthesis. Activating key enzymes in the mevalonate (MVA) synthesis pathway, such as HMG-CoA reductase (HMGCR), enables cholesterol biosynthesis. MVA is further modified to generate a variety of cholesterol for important biological processes such as membrane biosynthesis. Excess cholesterol is eliminated from the cell through ATP-binding cassette transporter A1 (ABCA1) [35]. In addition, low-density lipoproteins (LDLs) are taken up through membrane receptors (LDLRs), and high LDLRs levels promote LDL cholesterol-mediated breast cancer growth [36]. Reprogramming of cholesterol metabolism in tumors is mainly characterized by increased levels of intracellular cholesterol synthesis and abnormal metabolite accumulation [35]. This upregulation of cholesterol metabolism in both tumor cells and TME can promote oncogenic processes, such as tumor initiation, migration, and angiogenesis [37, 38].

Phospholipid (PL) synthesis, using DAG as a precursor, is enhanced in cancer, which regulates biological behaviors such as metastasis and drug resistance by modulating changes in membrane lipid composition and producing bioactive lipid second messengers [39]. Phosphatidylcholine (PC) is the predominant phospholipid in most cellular membranes. An increase in PC synthesis, along with elevated levels of choline cycle metabolites such as choline, phosphocholine (PCho), and glycerophosphocholine (GPC), has emerged as a significant hallmark of malignant transformation in tumors [40]. PC metabolic enzyme, choline kinase (ChoK), has been observed to be activated in various cancers [41]. Studies both in vitro and in vivo have shown that overexpression of ChoKα contributes to tumor progression, metastasis, and aggressiveness [42, 43].

In addition, PL catabolism is mediated by phospholipases (PLA2, C, and D), which can be recycled for PL biosynthesis and modulate various lipid-mediated signaling pathways promoting tumorigenesis. PL can also be hydrolyzed by PLC and PLD, producing DAG and phosphatidic acid (PA). This sustains the activity of key oncogenic signaling pathways involving PKC and mTOR [44]. Importantly, a significant portion of PL is hydrolyzed by PLA2, leading to the production of lysophosphatidylcholine (LPC) and AA. Subsequently, under catalysis of a series of enzymes, various lipid-derived mediators, including LPA, PGs, LTs, and S1P, are generated within tumor cells. LPC is catalyzed by lysophosphatidylcholine acyltransferases (LPCATs) to be reconverted into PC. Under the action of autotaxin (ATX), LPC is converted to LPA [45]. Similarly, AA participates in lipid mediator biosynthesis, producing prostaglandins (PGs) through cyclooxygenase (COX) and leukotrienes (LTs) through lipoxygenase (LOX). And sphingosine-1-phosphate (S1P) is also derived from sphingomyelin. These lipid-derived mediators are released extracellularly and act as crucial signaling molecules that mediate the crosstalk between the tumor and the TME for cancer progression [46].

#### Lipid storage in cancer

Increased uptake and endogenous synthesis of lipids in tumor cells lead to an increase in the cellular lipid pool. Acyl-CoA cholesterol acyltransferase (ACAT) converts free cholesterol to cholesteryl ester (CE) within the endoplasmic reticulum (ER) membrane, while excess intracellular FAs are ultimately converted into TAG by DGAT. These lipids are then stored as CE and TAG within lipid droplets (LD) in cells, reducing cell damage caused by peroxidation of free lipids within the cell [47, 48]. Lipids stored in LD can provide ATP response to metabolic stress by undergoing β-oxidation to produce acetyl-CoA. LDs serve as a critical reservoir of unsaturated FAs that cancer cells can use to maintain the function of cell membranes and organelles, particularly when there is an increased demand for lipids, such as during rapid oncogene-driven cell growth or a hypoxic environment [48]. Additionally, another key function of LDs is to protect cancer cells under ER stress and oxidative stress [49]. DGAT1, a key protein in lipid accumulation, promoting LDs formation and protecting cancer against lipid peroxidation, has been found to play indispensable oncogenic roles in melanoma and glioblastoma [50, 51].

#### Lipolysis in cancer

Degradation of TAG in LD can be initiated by adipose triglyceride lipase (ATGL), hydrolyzing TAG to produce DAG. DAG is then hydrolyzed by hormone-sensitive lipase (HSL) and monoacylglycerol lipase (MAL) to release FFA. Given that lipid synthesis and LD accumulation are common metabolic characteristics of cancers, ATGL, the key enzyme for LD mobilization, is generally downregulated in most cancer cells [52]. Recent research suggests that enhanced ATGL expression exerts an anti-tumor effect in triple-negative breast cancer cells [53]. In hypoxic cancer cells, LD is significantly accumulated, which is resulted by the activation of hypoxia-inducible gene 2 (HIG2) to inhibit ATGL-mediated lipolysis [54, 55]. However, ATGL upregulation in tumor exhibits pro-tumor effects in some adipose-infiltrated cancers, including colorectal and breast cancer [56, 57]. In coculture systems, breast cancer cells exhibit increased proliferation and migration after acquiring FAs from adipocytes, which is dependent on the lipolysis induced by ATGL in both adipocytes and cancer cells [57]. Besides, ATGL also play a crucial role in the development of colon cancer driven by obesity [58]. These studies indicate that lipolysis acts as a double-edged sword in cancer progression, and the underlying mechanisms require further elucidation.

#### Lipid oxidation in cancer

As FAs uptake and storage increase, FAs catabolism in cancer cells is often enhanced. The survival and metastasis of cancer cells also rely on the uptake and consumption of FAs. FAO serves as an energy source for tumor cells under nutrient-deficient conditions. Carnitine palmitoyl transferase 1 (CPT1), the rate-limiting enzyme involved in mitochondrial FAO of LCFAs, mediates the entry of FAs into mitochondria. Once FAs enter the mitochondrial matrix, they are oxidized to generate acetyl-CoA, which enters the TCA cycle to produce ATP. The long-chain acyl-CoA synthase (ACSL) enzyme family plays an important role in FAO and lipids biosynthesis, facilitating the production of fatty acyl-CoA [59]. Tumors exhibit high FAO activity by upregulating CPT1A expression. Moreover, upregulation of CPT1A expression can promote EMT and stemness, leading to the invasive and metastatic capabilities of cancer cells [60, 61].

Lipid peroxidation (LPO) is prone to occur in PUFA-phospholipids (PUFA-PLs), resulting in the accumulation of lipid peroxidation within cells, which is caused by an imbalance in the ratio of intracellular PUFA to MUFA. This phenomenon is commonly associated with an increase in PUFA due to LD synthesis inhibition or a decrease in MUFA due to downregulation of enzyme activity involved in MUFA synthesis. LPO is significant in mediating ferroptosis and is often inhibited in progressing tumors [62, 63]. LDs, essential mediators of free unsaturated FA (especially PUFA) storage, regulate LPO and susceptibility to ferroptosis [64]. Upregulation of DGAT promotes LD synthesis in glioblastoma and gastric cancer cells. Inhibiting the formation of LDs by silencing DGATs can induce LPO and ferroptosis, thereby inhibiting cancer cell metastasis [50, 65]. Maintaining MUFA-phospholipid (MUFA-PL) levels in the cell membrane is critical for tumor cells to avoid ferroptosis. ASCL3, which is upregulated in various cancers, mainly catalyzes MUFA generation to form fatty acyl-CoA, promoting the synthesis of MUFA-PLs [59]. Furthermore, ACSL4 promotes the increase of membrane PUFA-PL levels by acting on PUFA, an essential therapeutic approach for tumor by increasing LPO and inducing ferroptosis [66]. Inhibition of the key enzyme SCD1 in gastrointestinal cancers reduces MUFA production, inducing ferroptosis and exerting anti-tumor effects [67, 68]. As research progresses, inducing the accumulation of lipid peroxides and promoting ferroptosis have become potential targets for anti-tumor therapy through lipid metabolism.

### Oncogenic cues affecting tumor lipid metabolism

**Fig. 2:**
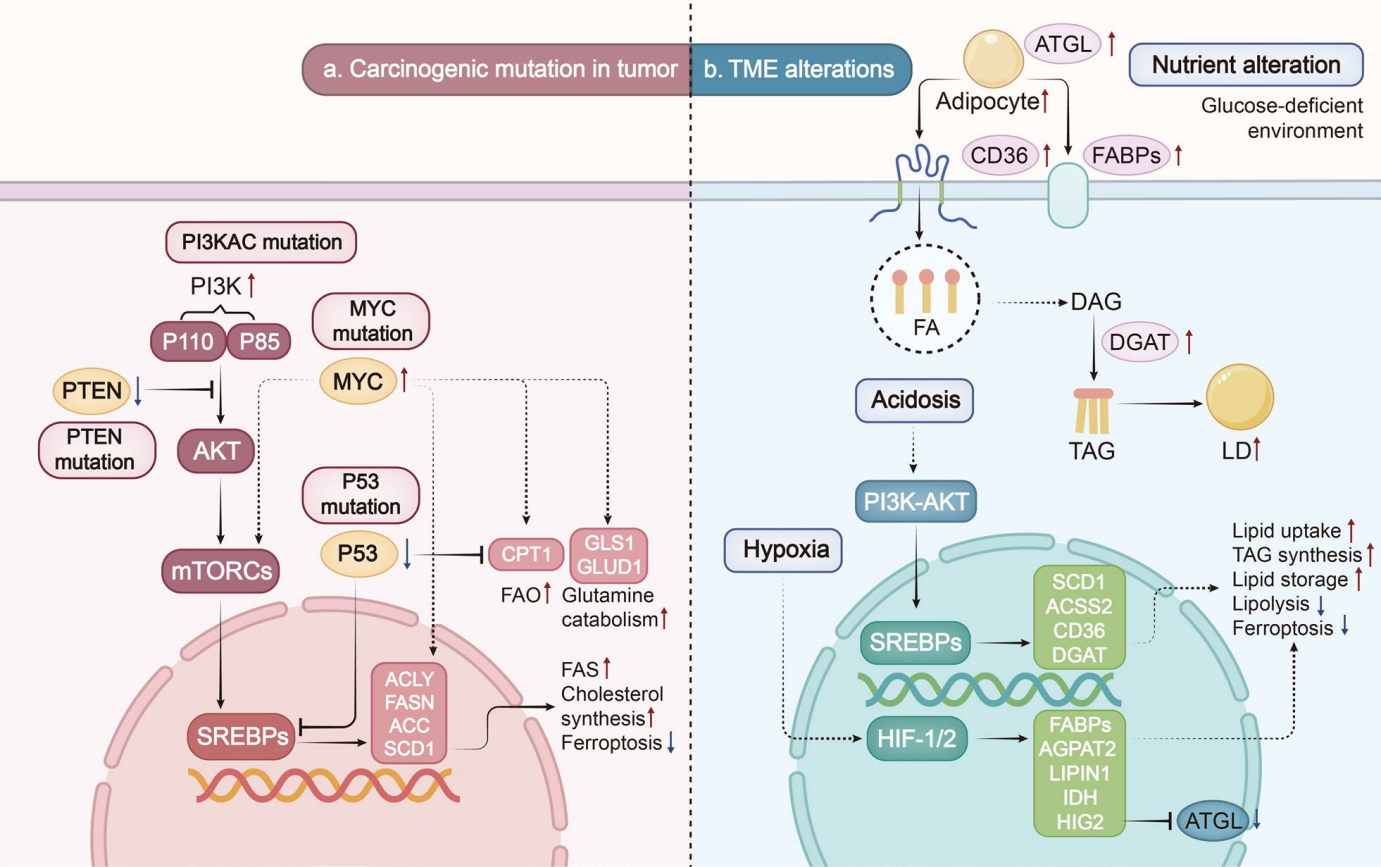
Lipid metabolic alterations induced by oncogenic events and TME cues. Alterations in lipid metabolism in tumor cells are driven by oncogenic events and tumor environmental cues, such as hypoxia and nutritional deficiency conditions. **a** Activating mutations in oncogenes (such as MYC) and inactivation of tumor suppressor genes (such as p53 and PTEN) often occur in various tumor cells. Alterations in these genes subsequently upregulate the expression of key enzymes involved in lipid metabolism by regulating the PI3K-AKT-mTOR signaling pathway to activate transcriptional activity of SREBPs, or acting directly on key enzymes. As a consequence, lipid metabolism in tumor cells is enhanced. **b** TME encompasses various factors such as hypoxia, acidosis, and nutrient deficiency that promote tumorigenesis and cancer progression through reprogramming lipid metabolism in tumors. Lipid uptake, synthesis, and intracellular lipid accumulation are significantly upregulated in TME by activating key signaling pathways and enzymes

Activation of oncogenes and loss-function of tumor suppressor genes are the main causes of tumorigenesis. They also play an important role in reprogramming tumor metabolism by regulating lipid metabolic enzyme expression [4]. Sterol regulatory element-binding proteins (SREBPs) act as key upstream regulators of lipid metabolism. SREBP is a transcription factor that promotes DNL by upregulating key enzymes such as ACLY, FASN, and SCD, which are closely linked to tumor proliferation, apoptosis, and invasion [69]. Moreover, SREBP maintains intracellular cholesterol levels by inducing LDL receptor-mediated cholesterol uptake and inhibiting ABCA1-mediated cholesterol export in a mTORC1-dependent manner [70]. Downstream lipid reprogramming events are induced by SREBPs and mutations in oncogenes such as PI3K and MYC, as well as tumor suppressor genes such as p53 and PTEN (Fig. 2a).

#### PI3K mutation

The dysregulation of the PI3K-AKT signaling pathway is a frequent occurrence in cancer and leads to metabolic reprogramming, where SREBPs play a crucial role as downstream regulatory targets. This pathway can be activated by various upstream signaling events, such as receptor tyrosine kinase (RTK) signaling or oncogenic mutations in PIK3CA [71]. By increasing lipid synthesis, this pathway promotes the occurrence and progression of liver cancer [72, 73]. The tumor suppressor PTEN is a critical negative regulator of the PI3K/AKT pathway, and its mutation can also activate this pathway [74]. Research has demonstrated that the loss of PTEN and the activation of PI3K/AKT lead to LD synthesis, contributing to prostate cancer progression [75]. In xenograft mice models of PIK3CA mutant breast cancer and PTEN-deficient prostate cancer, the excessive activation of the PI3K-AKT-mTOR signaling protects cancer cells from LPO and ferroptosis by SREBP1/SCD1-mediated MUFA synthesis [69]. Similarly, recent studies have shown that aspirin inhibits mTOR/SREBP-1/SCD1-mediated MUFA production and induces ferroptosis in PIK3CA mutant colorectal cancer [76]. In summary, the PI3K-AKT signaling may prove to be a potential therapeutic strategy for treating cancer as a metabolic disease.

#### P53 mutation

TP53, which encodes the tumor suppressor p53, is a commonly mutated oncogene in human cancers. p53 can bind directly to the promoter region of SREBP-1 and transcriptionally inhibit its expression, affecting downstream expression of key enzymes (ACLY, FASN) involved in lipogenesis [77]. Additionally, p53 suppresses the pentose phosphate pathway (PPP), which decreases NADPH production required for lipid synthesis [78, 79]. In breast cancer, p53 mutants have been shown to promote cancer progression by increasing cholesterol synthesis through enzymes involved in upregulation of the mevalonate pathway, highlighting the potential to target this pathway for p53-mutated tumors [80]. Moreover, the expression of genes key to FA synthesis (FASN, ELOVL6, and SCD1) is increased in p53-mutated tumors, while p53 transcription induces CPT1, which increases FAO and reduces intracellular lipid accumulation [81].

#### MYC mutation

MYC, a commonly activated oncogene in tumors, is known to promote transcriptional activation of genes involved in cell cycle, cell growth, and metabolism [82]. In addition to activating SREBP1, MYC directly regulates the expression of key enzymes involved in FA synthesis, such as ACLY, ACC, FASN, and SCD1, which have been shown to drive tumorigenesis in hepatocellular carcinoma (HCC) [83–85]. MYC also cooperates with SREBP2 to upregulate HMGCR for cholesterol metabolic reprogramming, contributing to the malignant phenotypes of tumor cells [86, 87]. Moreover, MYC-driven cancer cells exhibit enhanced glutamine utilization, with increased expression of key glutamine-metabolizing enzymes, including GLS1 and GLUD1, as well as the transporter protein SLC1A5 [88]. This augmented glutamine catabolism results in mitochondrial metabolic reprogramming to accommodate the replenishment requirements of the TCA cycle, supplying substrates for DNL, thereby sustaining cell vitality and growth [89]. What’s more, breast cancer cells with MYC overexpression show increased dependence on FAO for bioenergetics. Inhibiting FAO markedly diminishes the energy metabolism of these cells, suggesting that targeting FAO could be a potential therapeutic strategy for breast cancer [90, 91].

### Microenvironment factors affecting tumor lipid metabolism

Metabolic reprogramming of cancer cells is the result of a multifactorial process. Along with the activation of oncogenic signals caused by mutations in tumor cells, TME also plays a crucial role [92]. The TME encompasses various factors such as hypoxia, acidosis, and nutritional deficiencies, which promote tumor initiation and cancer progression by altering lipid metabolism in tumor cells (Fig. 2b).

#### Hypoxia

The rapid proliferation of solid tumors consumes a large amount of oxygen, leading to hypoxia as a typical feature of almost all TME [93]. The resulting hypoxia inhibits the pyruvate metabolic pathway of glucose, resulting in decreased citrate content in the TCA cycle. As citrate, the primary substrate for DNL, decreases, cancer cells turn to alternative carbon sources such as glutamate or acetate to produce acetyl-CoA for FA synthesis [2, 94]. Hypoxic tumor cells utilize glutamine and synthesize citrate under IDH1 catalysis, a process that relies on the expression of hypoxia-inducible factor (HIF1) [95, 96].

In addition to alterations in glucose metabolism, lipid metabolism also undergoes changes in hypoxic tumor cells. FA catabolism is dependent on oxygen, and tumor cells often inhibit FAO through various pathways. Hypoxia-activated HIF-1α and HIF-2α downregulate CPT-1 expression, which prevents FAs from entering the mitochondria for β-oxidation [97, 98]. As a result, FAs are redirected to LD storage, leading to increased lipid accumulation [99]. Additionally, HIF-1α upregulates FABPs expression in hypoxia, promoting FA uptake and lipid storage by regulating the expression of key enzymes involved in TAG synthesis [17, 100]. In clear cell renal cell carcinoma (ccRCC), hypoxia increases intracellular LD synthesis in a HIF-2α-dependent manner, which plays a crucial role in sustaining ER homeostasis and aggressive tumor behaviors [101]. Furthermore, HIF-1 upregulates the expression of low-density lipoprotein receptor-related protein 1 (LRP1) to promote lipoprotein endocytosis and the lipids storage in LDs, providing energy for cells during hypoxia [102, 103].

In summary, hypoxia induces genes involved in FA uptake, synthesis, and storage, leading to an overall increase in intracellular lipid content. The inhibition of key enzymes involved in FAO also supports lipid accumulation under hypoxia, and the accumulation of lipids in LDs helps sustain malignant behaviors of tumors [104].

#### Acidosis

TME is characterized by hypoxia and acidosis, both of which contribute to the metabolic reprogramming of tumor cells. Hypoxia results in lactate accumulation and H^+^ build-up, which alter the metabolic pathways of cancer cells and promote tumor metastasis [105]. Lactate activates the expression of the SLC1A5 and GLS1 to promote glutamine transport and catabolism, which provides substrates for the TCA cycle [32]. In the absence of acetyl-CoA produced by glycolysis in an acidic microenvironment, tumors rely heavily on FAO to generate energy by converting LCFAs into acetyl-CoA and producing NADH and FADH2 [106]. Interestingly, in tumor cells, an acidic environment upregulates CD36 and DGAT expression, promoting exogenous lipid uptake and the formation of LDs, thereby promoting metastasis through a TGF-β2-dependent mechanism [107]. Acidosis also stimulates fatty acid synthesis (FAS) and promotes hepatocarcinogenesis through activating the PI3K/AKT pathway, upregulating SCD1, and promoting its binding to peroxisome proliferator activated receptor-α (PPARα) [108]. Additionally, an acidic TME triggers SREBP2 activation in tumors, leading to upregulation of the downstream key enzyme ACSS2, which provides suitable growth conditions for cancer cells in the acidic environment. These findings suggest that an acidic TME and SREBP2 activation is associated with reduced overall survival of cancer patients [109].

#### Nutrient alteration

The rapid proliferation of tumors requires continuous acquisition of nutrients from the microenvironment, resulting in a nutrient-deficient TME[105, 110]. In obese phenotypes, changes in metabolic spectra within tumor tissues result in the accumulation of lipids in adipocytes within the TME [111, 112]. Consequently, tumor cells must alter their metabolic patterns to enhance the utilization of lipids for energy supply to sustain their biological behaviors. In fat-rich TME, cancer cells can produce cytokines that induce ATGL-dependent lipolysis in the adipocytes surrounding the tumor, resulting in the release of FFAs [113]. These FFAs are then taken up by cancer cells via CD36 and FABP3/4 to form LDs, which act as energy sources for malignant cells. Moreover, lipid transfer from adipocytes to cancer cells can also be facilitated by extracellular vesicles (EVs) [114, 115]. Adipocytes in the TME have been shown to act as metabolic regulators that promote the growth and survival of colon cancer cells [116]. When co-cultured with breast cancer cells, the consumption of TAGs within adipocytes is increased, which in turn transfers adipocyte-derived FFAs to breast cancer cells, increasing CPT1A levels and driving FA metabolism in cancer cells [117].

### Dietary factors affecting tumor lipid metabolism

Dietary interventions can change metabolite levels in the TME, which may then affect cancer cell metabolism to alter tumor growth [118]. Obesity and excessive high-fat diets (HFDs) are associated with increased overall and cancer-specific mortality, especially among patients with breast, colon and uterine cancer [119]. Excessive adipose expansion during obesity causes adipose dysfunction and inflammation to increase systemic levels of proinflammatory factors. Cancer-associated adipocytes can enter the TME to enhance pro-tumor effects [120]. In obese mouse models, HFDs increase the number of LGR5^+^ intestinal stem cells and activate the lipid metabolism transcription factor PPARγ for their tumorigenic potential [121]. Importantly, fat-mediated inflammatory signaling and the immunosuppressive microenvironment in tumors are the main causes of tumor metastasis [122]. For instance, HFD-induced obesity leads to CD8^+^ T cell exhaustion by reducing the production of granzymes and cytokines (IFN-γ and TNF-α), ultimately accelerating tumor growth in mouse models [123, 124]. Moreover, HFDs can induce lipid accumulation in prostate tumors in a SPREP-dependent manner, facilitating metastasis in mouse models [125]. Similarly, chronic HFDs alter biological behaviors, including angiogenesis and proliferation, in breast cancer [126].

Notably, different types of dietary lipids exhibit heterogeneity in driving the biological behavior of tumors. A study demonstrates that dietary palmitic acid, in contrast to OA or LA, enhances metastasis in oral carcinomas and melanoma in mouse models [127]. SFA intake is associated with an enhanced MYC signaling and poorer outcome in prostate cancer patients [128]. OA can also favor survival and chemotherapy resistance in gastric cancer [129]. Mechanistically, HFDs raise systemic FA levels, including SFAs and unsaturated FAs, which further enhances FAO, producing enough energy to facilitate tumor progression. In addition, two essential FAs: ALA (ω-3 PUFA) and LA (ω-6 PUFA), play proinflammatory and anti-inflammatory roles in tumors, respectively. In patients with metastatic colorectal cancer, primary tumors exhibit significantly elevated levels of ω-6 PUFAs and reduced levels of ω-3 PUFAs compared to those in patients with non-metastatic cancer [130]. Dietary LA stimulates invasion and peritoneal metastasis of gastric cancer through COX-1-catalyzed lipid metabolism [131]. Consequently, alterations in dietary lipid consumption, adopting a low-fat eating pattern or increasing ω-3 PUFAs intake, may represent a selective anti-tumor therapy.

## Landscape and mechanisms of lipid metabolic reprogramming in tme

**Fig. 3:**
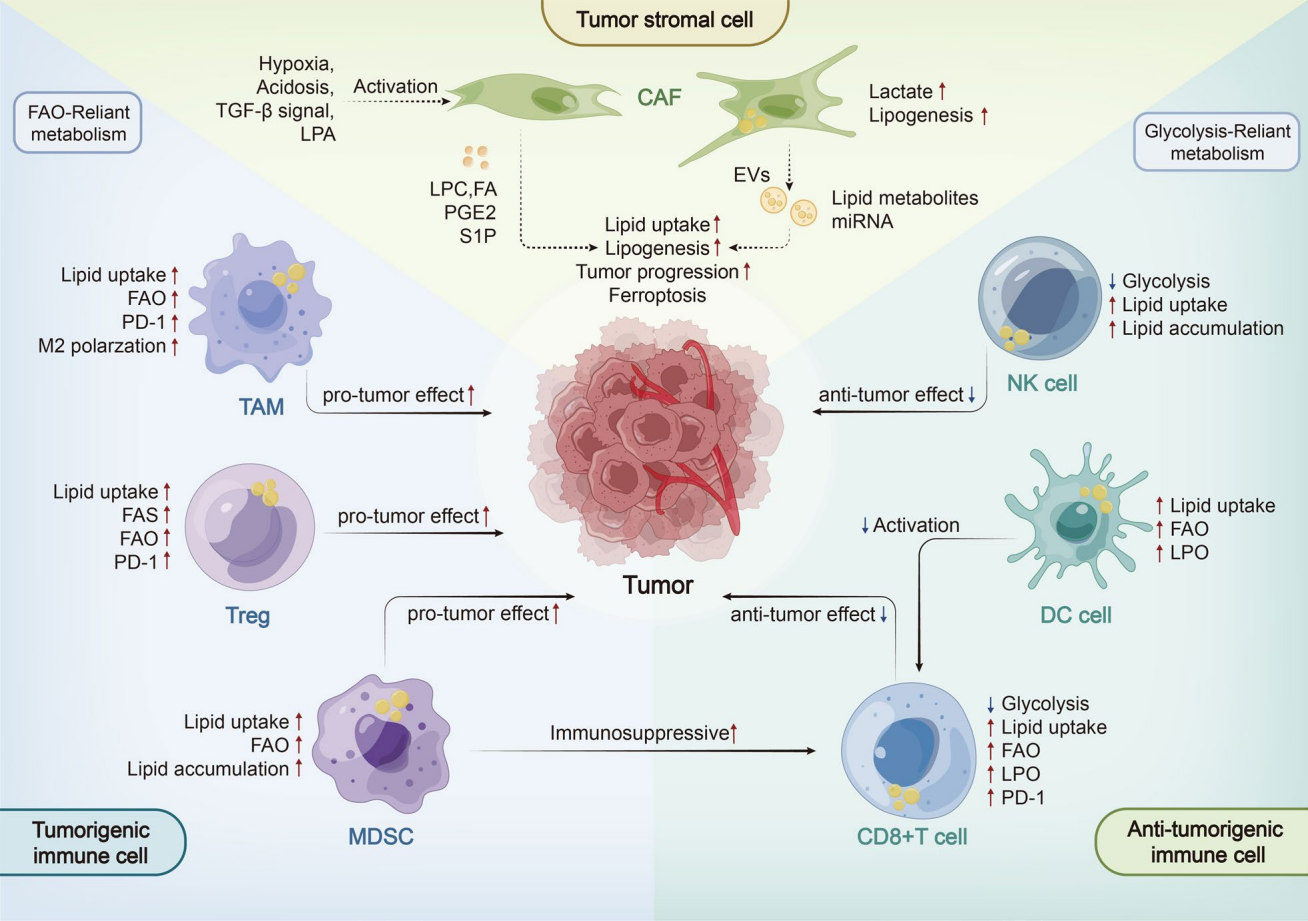
Lipid metabolism landscape in TME. Tumor cells have the ability to educate TME cells into a pro-tumor phenotype by secreting metabolites and signaling molecules, such as cytokines and bioactive lipids, to TME, which further facilitates cancer progression. For instance, CAFs activated by tumor cells lead to increased levels of lipid synthesis, which serve as an essential energy source for tumor cells and induce the formation of an immunosuppressive microenvironment through secreting EVs and signaling molecules. Besides, tumor-infiltrating CD8^+^ T cells and NK cells also exhibited increased lipid uptake and FAO. Similarly, lipid accumulation in TAMs also regulates their polarization and functional phenotypes. Increased lipid uptake and FAO in immunosuppressive cells, such as Tregs and MDSCs, further enhanced their pro-tumor effects. Taken together, lipid metabolic reprogramming facilitates crosstalk between tumor cells and TME cells, fueling tumor cells and changing functional phenotypes of TME cells

As cancer progresses, the TME also undergoes lipid metabolic reprogramming. It is worth noting that tumor cells play a significant role in modifying TME (e.g., acidosis, lipid accumulation) by producing metabolites and lipid-related signaling molecules. This in turn influences metabolic patterns and immune phenotype of TME cells, resulting in immune microenvironment remodeling [65]. For example, the secretion of lipids by CAFs in the TME promotes tumor progression by directly supplying energy sources to tumor cells. What’s more, lipid metabolic reprogramming in CAFs also influences its own cytokine secretion function, which subsequently modulates the immune responses and promotes the formation of an immunosuppressive microenvironment. In addition, alterations in lipid metabolism patterns in immune cells also favor the construction of an immunosuppressive microenvironment, supporting tumor immune escape. Therefore, tumor progression is the result of a co-evolutionary process between tumor and TME. Moving forward, this section will focus on lipid metabolic alterations in TME cells and their interactions with tumor cells (Fig. 3).

### Lipid metabolic reprogramming in CAFs

Cancer-associated fibroblasts (CAFs), an important stromal cell type in the TME, are activated by TGF-β and LPA signaling in TME [132, 133]. They synthesize and secrete lipids and bioactive lipid signaling molecules, playing an essential role in tumor metabolic alteration, proliferation, invasion and immune responses [134]. To adapt to the TME, CAFs undergo lipid metabolic reprogramming characterized by an increase in lipid synthesis, storage, and secretion by upregulating key enzymes like FASN and SCD [135, 136]. This constructs a microenvironment with lipid accumulation, leading to metabolic reprogramming and biological behavior enhances in tumors [115, 137].

In the hypoxic and nutrient-poor microenvironment, CAFs upregulate SCD1 expression via HIF-1α, increasing the abundance of lipids such as OA in CAFs, resulting in promoting lung cancer growth [136, 138]. Therefore, targeting SCD1 in CAFs could be a promising therapeutic strategy. Recent metabolomics studies have shown that CAF-derived lipids enhance lipid uptake in tumor cells, promoting peritoneal metastasis of colorectal cancer [139]. This phenomenon also depends on the upregulation of CPT1 in CAFs, which enhances FAO and shapes the TME for colorectal cancer metastasis [140].

In addition, CAFs shape lipid metabolism and biological behavior of tumor cells by secreting biologically active lipid molecules and EVs containing small molecules and lipid metabolites [20, 141, 142]. Overexpressed biologically active lipids, such as LPC, in CAFs can be released into the TME and absorbed by tumor cells to promote tumor proliferation and migration through intracellular lipid metabolic reprogramming [135, 143]. Mechanistically, LPC is hydrolyzed by LPA in cancer cells, activating the AKT signaling pathway [144]. PGE2 and S1P in CAFs also play vital roles in tumor progression in breast cancer and neuroblastoma [145–147]. What’s more, a high concentration of proinflammatory cytokines secreted by CAFs supernatant can induce upregulation of cholesterol metabolism in prostate cancer cells, promoting androgen receptor therapy resistance [148]. miR-522, secreted from CAF-derived EVs, inhibits ferroptosis in cancer by targeting arachidonate lipoxygenase 15 (ALOX15) and blocking LPO [149]. In breast cancer, CAF enhances their exogenous lipid uptake capacity by inducing upregulation of FATP1 [150, 151]. Similarly, CAFs promotes colon cancer cells to absorb lipids secreted from CAFs through CD36, thereby promoting cancer cell migration [135]. Lactate secreted by CAF induces lipid metabolic reprogramming in prostate cancer, leading to the LD formation and mobilization, concurrently enhancing tumor invasiveness [31]. These evidences suggest that the signaling molecules and metabolites secreted by CAFs play an essential role in tumor progression and may become promising therapeutic targets in future [152].

Overall, the crosstalk between CAFs and cancer cells is mediated by lipid metabolic reprogramming that contributes to cancer progression, metastasis, and therapeutic resistance. Moreover, recent studies have shown that lipid metabolic reprogramming of CAFs also plays an important role in remodeling the tumor immune microenvironment. For example, CD36^+^ CAFs recruit MDSCs through upregulating MIF expression, thus promoting immune escape in HCC. Inhibitors targeting to CD36 can restore the anti-tumor immune response in HCC and synergistically enhance the anti-tumor effect of anti-PD-1 therapy [153].

### Lipid metabolic reprogramming in immune cells

Lipids are critical metabolites that support the biological activities of immune cells. Under normal conditions, immune cells with anti-tumor activity, such as effector CD8^+^ T cells, NK cells, and M1 macrophages, depend on glycolysis for their maturation and function, whereas immune-regulatory cells such as Tregs, M2 macrophages, and MDSCs rely on FAO to exert their tumor immune suppression effects [115]. When tumors occur, lipid metabolism of tumor cells and stromal cells, such as adipocytes and CAFs, contributes significantly to the establishment of a TME characterized by low glucose and high lipid accumulation [115, 137, 154]. In such a TME, immune cells display increased immunosuppressive effects by regulating their lipid metabolism patterns, and subsequently promoting tumor progression [65]. For instance, lipid accumulation and enhanced FAO in TAMs contributed to its polarization to the M2 phenotype, which blocked anti-tumor T cell responses and supported the immunosuppressive function of T cells [155]. Increased uptake of oxidized lipids and enhanced lipid peroxidation in CD8^+^ tumor-infiltrating lymphocytes (TILs) lead to their immune dysfunction, whereas lipid peroxidation resolution restored the functionalities of CD8^+^ TILs in vivo [11] (Table 1).

**Table 1:** Lipid metabolism in immune cells

Immune cell	Lipid metabolic alteration	Effect on immune cells	Mechanism	Treatment Strategy	Reference
CD8^+^ T cells	FAO↑	Maintained anti-tumor function through FAO upregulation	Promoted switch from glycolysis to FAO by PD-1; Activated the expression of PPAR, STAT3 signaling	Promoting FAO and related signals; Inhibiting metabolic conversion through PD-1 blockade	[162–166]
	Lipid uptake↑ LPO↑	Impaired CD8^+^ T cell function for tumor progression	CD36 upregulation; Increased lipid accumulation for ferroptosis	Targeting CD36; Degrading lipid peroxides	[11, 161, 169–171]
Treg cells	Lipid uptake↑	Enhanced the immunosuppression effect	CD36 upregulation;	Targeting CD36	[8, 175]
	FAS↑ FAO↑	Elevated the level of Treg cell Enhanced the immunosuppression effect	Activated the expression of SREBP, PI3K and PD-1; FASN upregulation;	Targeting SREBP and PD-1	[175–179]
TAMs	Lipid uptake↑ FAS↑	Released pro-tumorigenic cytokines Enhanced the immunosuppression effect	CD36 upregulation; Increased LD accumulation	Targeting CD36	[155, 187–190, 192–194]
	FAO↑	Increased M2-like polarization; Enhanced the immunosuppression effect	Activated the expression of PPAR signaling; Enhanced the rate of FAO	Targeting FAO	[9, 187, 188, 196]
DCs	Lipid uptake↑ FAO ↑	Reduced the ability of T cell stimulation	Atg5 downregulation; CD36 and CPT1 upregulation;	Targeting FAO and CD36; PD-1 blockade	[199–201, 203]
	LPO↑	Impaired DC function for tumor progression	Activated the expression of XBP1; Lipid peroxide accumulation	Degrading lipid peroxides	[202]
NK cells	Lipid uptake↑ LPO↑	Impaired NK cell function for tumor progression	CD36 upregulation; Activated the expression of PPAR signaling; Increased lipid peroxide accumulation	Degrading lipid peroxides	[12, 207–210]
MDSCs	Lipid uptake↑	Enhanced the immunosuppression effect	FATPs, VLDLR and CD36 upregulation; Activated the expression of STAT3;	Targeting FTAPs and CD36	[10, 218–221]
	FAO↑	Enhanced the immunosuppression effect	CPT1 upregulation	Targeting FAO	[222]

#### ***CD8***^+^***T cells***

**Fig. 4:**
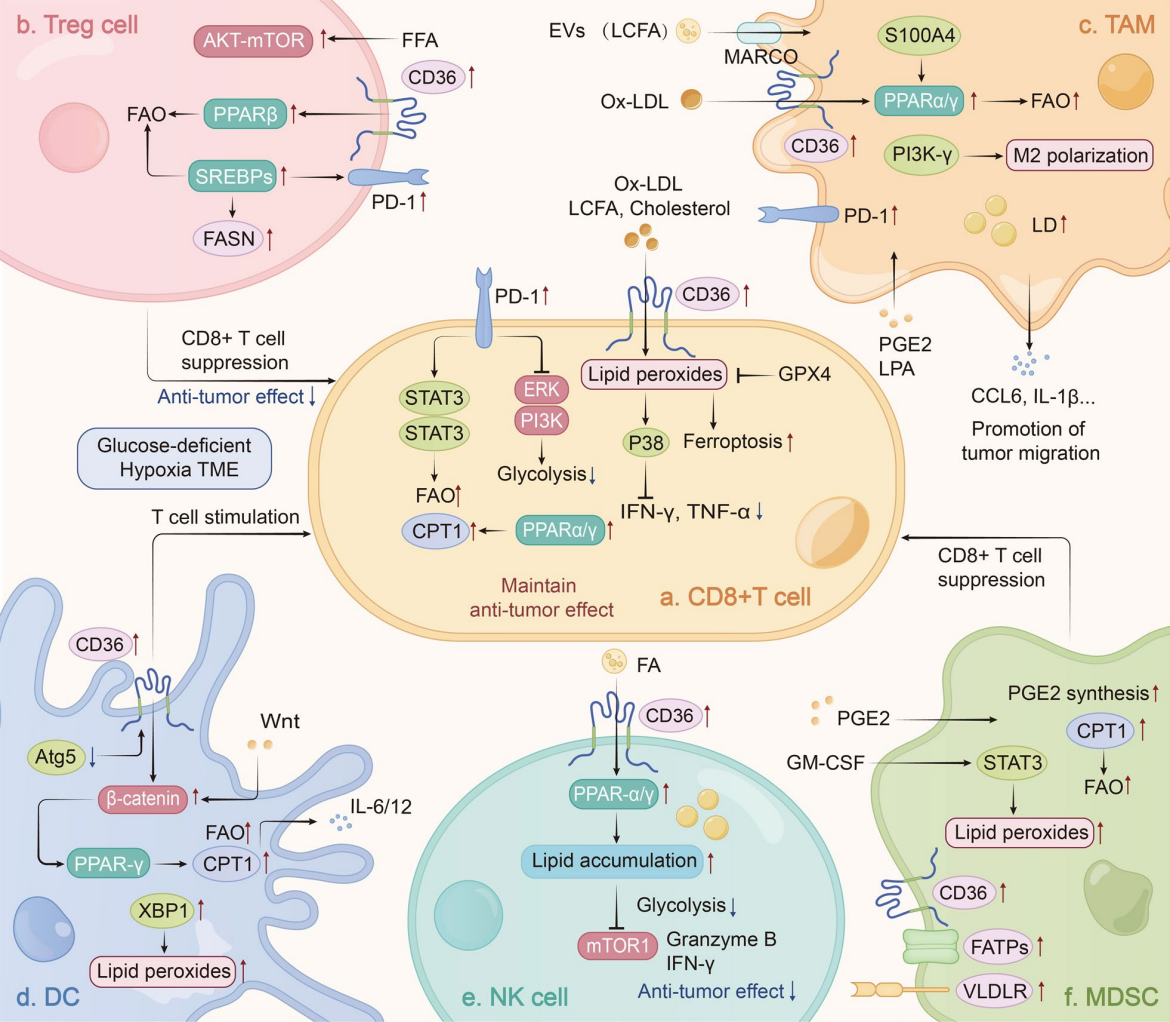
Mechanism of lipid metabolic reprogramming in immune cells. A hypoxic, glucose-deficient, lipid-rich TME often educates immune cells into an immunosuppressive and pro-tumor phenotype by reprogramming their lipid metabolism. Immune cells in the TME undergo lipid metabolism reprogramming by directly absorbing excess lipids in the TME or enhancing their own lipid uptake, synthesis, and oxidation. Overexpression of PD-1 and CD36 on CD8^+^ TILs promotes metabolic transition by activating STAT3 or PPARs, which results in enhanced FAO (**a**). In Tregs cells, lipid metabolism is enhanced by CD36-PPARβ signaling, AKT-mTORC1 signaling, and SREBPs-mediated overexpression of FASN (**b**). TAMs can use lipids directly in TME or uptake exosomes containing LCFAs. FAO in TAMs is also enhanced by CD36-PPARs signaling (**c**). In DCs, NK cells, and MDSCs, CD36-PPARs signaling also plays a critical role in modulating their lipid metabolism (**d**–**f**)

CD8^+^ TILs are an important component of anti-tumor immune cells, but their cytotoxic effects change to an exhausted state as cancer progresses [156–158]. In response to the limited nutrients and glucose in the TME, CD8^+^ TILs experience a significant reduction in glycolytic activity. To sustain their anti-tumor functions, CD8^+^ TILs undergo metabolic adaptation and promote FAO as an alternative energy source [159, 160]. However, excessively elevated lipid metabolism can lead to lipid peroxidation and ROS accumulation within the cell, further impairing their anti-tumor effects [161] (Fig. 4a).

CD8^+^ TILs often undergo metabolic transitions to adapt to the hypoxia, glucose deficiency and lipid accumulation in the TME. This transition involves converting glycolysis to FAO, maximizing their activity to maintain their anti-tumor function. For instance, in a mouse model of obesity-associated breast cancer, CD8^+^ TILs downregulate glycolytic activity and enhance FAO [162]. Additionally, CD8^+^ T cells in MC38 colorectal cancer cell line and B16 melanoma cell line-bearing mice models show an FAO increase with the upregulation of CPT1 [163–165]. PD-1 signaling plays a crucial role in this process by affecting the PI3K and ERK pathways [166]. Enhanced PD-1 signaling in CD8^+^ TILs promotes metabolic transition by STAT3 activation-mediated upregulation of FAO [162]. Similarly, the IL-9/STAT3 signaling maintains the anti-tumor effect by upregulating FAO activity, decreasing intracellular LPO and resisting ferroptosis in CD8^+^ Tc9 (cytotoxic T lymphocyte subset 9) cells [165]. In addition, PPAR signaling also activates FAO in CD8^+^ TILs. When TME changed to a state of hypoxia, glucose deficiency, or lipid accumulation, the PPARα signaling in CD8^+^ TILs is activated, and the FAO within the cell is enhanced [163]. The PPAR agonist bezafibrate upregulates CPT1 to increase FAO, leading to enhanced anti-tumor effects during anti-PD-1 therapy in animal models [163, 164]. Interestingly, elevated tumor-intrinsic lipid metabolism can competitively inhibit T cells, leading to a decrease in lipid metabolism levels [167, 168]. These findings highlight that an appropriate increase of FAO in CD8^+^ TILs can enhance their cytotoxic effects, especially when combined with anti-PD-1 therapy.

Lipid accumulation is a common metabolic alteration in the TME. Extensive studies have shown that excessive lipid accumulation is observed in exhausted CD8^+^ TILs, where the fatty acids receptor is always upregulated. Specifically, CD36 expression on CD8^+^ TILs has been linked to tumor progression and poor survival rates in cancer patients [169]. In mice models of melanoma, colorectal cancer, and pancreatic ductal adenocarcinoma (PDAC), CD8^+^ TILs have been reported to upregulate CD36 expression in order to facilitate extracellular lipid uptake [11, 169, 170]. However, excessive lipid accumulation can lead to elevated levels of intracellular cholesterol, LCFA, and ox-LDL, which in turn resulting in CD8^+^ T cell exhaustion. Accumulated lipids, particularly oxidized low-density lipoprotein (ox-LDL), can increase intracellular LPO levels, ultimately resulting in ferroptosis and reducing the production of anti-tumor factors such as IFN-γ and TNF-α by activating p38, thereby impairing anti-tumor immune responses [11, 169]. Furthermore, LCFAs accumulation in CD8^+^ T cells can impair mitochondrial function and reduce FAO, thereby suppressing the cytotoxic effects of CD8^+^ T cells in PDAC [170].Increased cholesterol levels in CD8^+^ T cells are also strongly associated with its functional exhaustion [161, 171], resulting from increased PD-1 and ER stress [161]. Therefore, inhibiting lipid accumulation in CD8^+^ T cells, such as degrading lipid peroxides by glutathione peroxidase 4 (GPX4), is a feasible strategy for sensitizing immunotherapy [11].

Intriguingly, in contrast to the previous findings, ACAT1 inhibition has been shown to enhance the anti-tumor effect of CD8^+^ T cells by increasing free cholesterol in the cytoplasm, which promotes membrane synthesis [172]. These results, along with other studies, suggest that the impact of cholesterol and other lipids on CD8^+^ T cell function is largely dependent on their intracellular levels. Taken together, it can be concluded that optimal levels of lipids are necessary to maintain intracellular FAO and biological function, thereby sustaining the effector state of CD8^+^ T cells, whereas excessive levels of lipids can lead to T cell exhaustion in the TME.

#### Treg cells

Regulatory T (Treg) cells in the TME dominate in weakening anti-tumor responses through secreting cytokines and expressing cell surface inhibitory receptors [173]. In fact, the overall lipid metabolism in Treg cells is upregulated, including lipid uptake, FAS, and FAO, which ultimately contributes to the immunosuppressive effect [174] (Fig. 4b).

Increased lipid uptake through the upregulation of CD36 is a prominent feature of Treg cells in the TME. This has been observed in mice models of colorectal cancer, melanoma, and glioblastoma, where upregulation of CD36 in Treg cells promotes lipid metabolism under environmental stresses [8, 175]. CD36-mediated intracellular lipid accumulation activates the PPARβ signaling pathway, which regulates mitochondrial metabolism and further enhances the immunosuppressive function of Treg cells in tumor [8]. Therefore, targeting CD36 in Treg cells can be an effective approach to restore effective anti-tumor immune responses.

Increased lipid synthesis, including FA and cholesterol, is the predominant metabolic alteration in tumor-infiltrating Treg cells, which is facilitated by the action of SREBPs and their target genes [176]. FASN-mediated DNL contributes to the functional maturation of Treg cells, while PD-1 expression in tumor-infiltrating Treg cells is also elevated, dependent on SREBP activation [176, 177]. Additionally, Treg cells rely on FAO for oxidative phosphorylation to fulfill their functions [175, 178]. Specifically, Treg cells enhance FA and cholesterol metabolism via AKT-mTORC1 signaling, promoting cell proliferation and inducing the expression of critical immune suppression molecules, including CTLA-4 and ICOSs [179]. Consequently, targeting lipid metabolism in Treg cells, such as CD36 and SREBP, may be a promising way to enhance the efficacy of cancer immunotherapy, worthy of further study [8, 176].

#### TAMs

Tumor-associated macrophages (TAMs), which represent another important component of TME, mainly rely on lipids metabolism to maintain their differentiation and function. TAMs are primarily divided into anti-tumor M1 and pro-tumor M2 phenotypes, M1 macrophages use fatty acids to synthesize inflammatory mediators and obtain most ATP from aerobic glycolysis, while M2 macrophages enhance FAO by fuel fatty acids to obtain ATP [180]. The TME contains various signaling molecules that can alter lipid metabolism in TAMs, including increased lipid uptake, FAS, and FAO, which promote TAM polarization to the pro-tumor M2 phenotype. This polarization exerts immunosuppressive effects, promoting tumor growth, metastasis, and angiogenesis [181] (Fig. 4c).

Lipid metabolites or signaling molecules in TME regulate differentiation and polarization of macrophages, exerting immunosuppressive effects [182]. Inflammation-related cancers exhibit increased levels of PGE2 and LPA, which promote the differentiation of monocytes into immunosuppressive TAMs phenotype [183, 184]. Furthermore, tumor cells and adipocytes release significant amounts of lipid-rich exosomes to TME, leading to the differentiation of TAMs toward the M2 phenotype [185, 186].

Lipid transport proteins, particularly CD36, play a crucial role in lipid accumulation within TAMs, which further affect the activation of TAMs [187, 188]. In liver metastasis mouse models, CD36 was upregulated in tumor-infiltrating TAMs [189], and CD36 knockout resulted in decreased lipid accumulation in TAMs [187]. Moreover, the elevated macrophage receptor for collagenous structures (MARCO) on TAMs also promotes lipid accumulation, which is induced by IL-1β in prostate cancer cells. CD36 in TAMs mainly mediates extracellular lipid uptake, especially ox-LDL, and the internalization of tumor cell-released lipids in EVs [155, 189, 190]. Lipid-loaded TAMs then release pro-tumorigenic cytokines, including CCL6, to TME, and finally promotes cancer cell migration [190]. Mechanically, lipid accumulation in TAMs leads to increased expression of PI3K-γ, which play a critical role in TAM polarization [155]. Loss of CD36 in TAMs can restore cytotoxic effects of CD8^+^ T cells and inhibit the growth of metastatic tumors in liver [189].

In addition to lipid uptake, TAMs also exhibit a phenotype with enhanced lipid biosynthesis, which contributes to the secretion of tumor-promoting cytokines and ROS response. Evidence has shown that inhibiting FASN in TAMs can significantly reduce extracellular tumor-promoting cytokines and hinder tumor progression [191]. In addition, continuous FAS activation induced by SERBPs is a major cause for maintaining the activity of M2-like TAMs in the Treg-mediated immunosuppressive microenvironment [192]. Activation of lipid uptake and synthesis promotes the formation of LDs, which further enhances their tumor-promoting functions. Inhibiting LD formation leads to a shift in TAM phenotype from M2 to M1, which may be an effective strategy to counteract TAM-mediated immunosuppression [193, 194].

An increased FAO is also very common in TAMs, which helps maintain their pro-tumorigenic functions. M2 macrophages rely on enhanced FAO instead of glycolysis for energy. High levels of FAO promoted mitochondrial oxidative phosphorylation, production of ROS, and phosphorylation of STAT6, promoting activation and transcription of genes that regulate TAMs generation and function [187]. For example, FAO is critical for M2-induced HCC migration, increasing ROS and secretion of IL-1β [195]. Mechanistically, the high rate of FAO in M2 macrophages attributes to the activation of the PPARγ [9, 188, 196]. TAMs exhibit downregulated expression of RIPK3 in HCC, which significantly inhibits caspase1-mediated PPAR cleavage[9]. Furthermore, elevated S100A4 can significantly activate PPARγ and increase FAO levels [188]. Inhibiting FAO or PPARγ signaling pathway in TAM impedes their polarization toward a pro-tumorigenic M2 phenotype and inhibits tumor progression [187, 196]. Therefore, inhibiting FAO may be a useful way to modulate TAMs polarization to an anti-tumorigenic phenotype, thus enhancing anti-tumor immune responses.

Targeting M2-like TAMs also presents a promising therapeutic strategy for cancer treatment. Compared to M1-like TAMs, M2-like TAMs express higher levels of PD-1. Therefore, combining anti-PD-1 therapy with lipid metabolism inhibitors may selectively attack M2-like TAMs, improving the effectiveness and sensitivity of therapy [20, 197].

#### DCs

Dendritic cells (DCs) are the primary antigen-presenting cells that activate CD8^+^ T cells and mediate anti-tumor immune responses. Lipid metabolic alterations of DCs in TME, such as increased lipid uptake, lipid accumulation and enhanced FAO, can decrease their antigen presentation capacity and facilitate immune evasion in tumors [198] (Fig. 4d).

Increased lipid uptake in DCs leads to lipid accumulation and FAO promotion, which impairs their ability to stimulate T cells [199, 200]. In tumor-bearing mice, an upregulation of fatty acids receptor is observed in DCs within the tumor, resulting in a significant increase in intracellular lipid levels compared to peripheral blood DCs [199]. Research has found that increased CD36 expression was found in DCs in TME due to the absence of the autophagy gene Atg5. Restoring Atg5 expression and blocking CD36 in DCs may effectively reduce lipid accumulation [201]. Moreover, lipid-laden DCs have a profound defect in their ability to process and present soluble antigens [199]. Similarly, XBP1-mediated lipid peroxide accumulation in tumor-associated DCs can induce ER stress and inhibit antigen presentation function of DCs, thus indirectly hindering its anti-tumor effect [202]. Elevated FAO level mediated by CPT1A is also an essential metabolic feature of tumor-associated DCs, which ultimately leads to the secretion of immune-suppressive cytokines, such as IL-6 or IL-12, promoting Treg cell aggregation to TME. Mechanically, intracellular β-catenin-PPARγ activated by extracellular Wnt5a contributed to this process [203]. Blocking lipid accumulation and FAO in tumor-associated DCs may enhance their anti-tumor efficacy.

#### NK cells

Natural killer (NK) cells, a particular type of T cells, play a crucial role in protective immunity against tumors and viral infections. They break infected cells or tumor cells via secreting perforin and granzyme [204]. Acidosis and nutrient-deficient TME are associated with the dysfunction of NK cells [205, 206]. Similarly, lipid-rich microenvironments let lipid accumulated within NK cells, ultimately impairing their anti-tumor functions (Fig. 4e).

Lipid accumulation in NK cells is the main cause of its dysfunction for killing tumor cells. Notably, an increased lipid accumulation and high CD36 expression in splenic NK cells isolated from surgery-treated tumor-bearing mice were observed [12]. Similarly, another study reported that individuals with obesity were deficient in NK cell numbers compared to lean individuals [207]. Besides, NK cells can directly take up lipids-rich EVs secreted from lung mesenchymal cells, which leads to lipid accumulation within the cells [208]. As a consequence, lipid-laden NK cells exhibit decreased production of granzyme B and IFN-γ, which ultimately results in a diminished anti-tumor effect [12, 207, 209]. In particular, increased FA levels in the lymphoma environment are also found to be related to NK cells dysfunction [209]. Further study indicated lipid accumulation in NK cells is driven by PPARα/δ signaling pathway under lipotoxic obese environment. Inhibiting PPARα/δ or blocking the lipids transportation can reverse NK cell metabolic paralysis and restore its cytotoxicity [207]. However, recent research on HCC suggested a potentially beneficial role for cholesterol accumulation in NK cells, as it promotes membrane lipid rafts formation and enhances anti-tumor effects [210].These findings are controversial as to the role of different lipids in NK cells, requiring more high-quality research to explore.

#### MDSCs

Myeloid-derived suppressor cells (MDSCs), divided into mononuclear MDSCs (M-MDSC) and polymorphonuclear MDSCs (PMN-MDSC), play an important role in shaping the immunosuppressive microenvironment [211]. It has been well demonstrated that increased lipid uptake and FAO in MDSCs can promote its production of immune-suppressive cytokines that inhibit cytotoxic effects of CD8^+^ T cells [212] (Fig. 4f).

Bioactive lipid signaling factors in TME promotes the realization of immune cells function. Specifically, in breast and colon cancer, studies have identified PGE2 as a crucial factor in activating MDSCs in TME [213, 214]. Mechanistically, tumor-derived PGE2 induces nuclear accumulation of p50 NF-κB in M-MDSCs, leading to NO-mediated immune suppression [215]. Therefore, blocking PGE2 has been proposed as a therapeutic strategy to prevent immune suppression of MDSCs and restore its anti-tumor effects in TME [215, 216].

In addition to lipid signaling factors, lipid metabolic reprogramming of MDSCs, characterized by increased lipid uptake and FAO, is essential in regulating their functions. Importantly, exogenous FA uptake promotes their ability to suppress the activity CD8^+^ T cell, facilitating tumor progression [211, 217, 218]. Notably, unsaturated FAs have a stronger effect on MDSC suppression, as MDSCs treat with LA exhibited more substantial suppression than those treated with palmitic acid [219]. CD36, FATPs, and VLDLR expression are upregulated in tumor-activated PMN-MDSCs. Inhibition of CD36 can reduce the immunosuppressive function of MDSCs [218]. Similarly, FATP2 can also be overexpressed in PMN-MDSCs, induced by tumor cell-derived GM-CSF and the activation of STAT3 signaling [10, 220]. It promotes intracellular PGE2 synthesis and exerts tumor-promoting effects [220]. Inhibiting FATP2 in MDSCs enhances anti-PD-L1 tumor immunotherapy by upregulating CD107a and decreasing PD-L1 expression on CD8^+^ TILs [221]. In addition, Mouse models showed that tumor-infiltrating MDSCs had increased FAO levels, along with upregulation of CPT1, and increased oxygen consumption. Inhibition of FAO combined with low-dose chemotherapy can restrain the immunosuppressive effects of MDSCs and induce significant anti-tumor effects [222]. Therefore, targeting the lipid metabolism of MDSCs may sensitize the effects of cancer immunotherapy.

## Strategies for tumor comprehensive therapy by targeting lipid metabolism

**Fig. 5:**
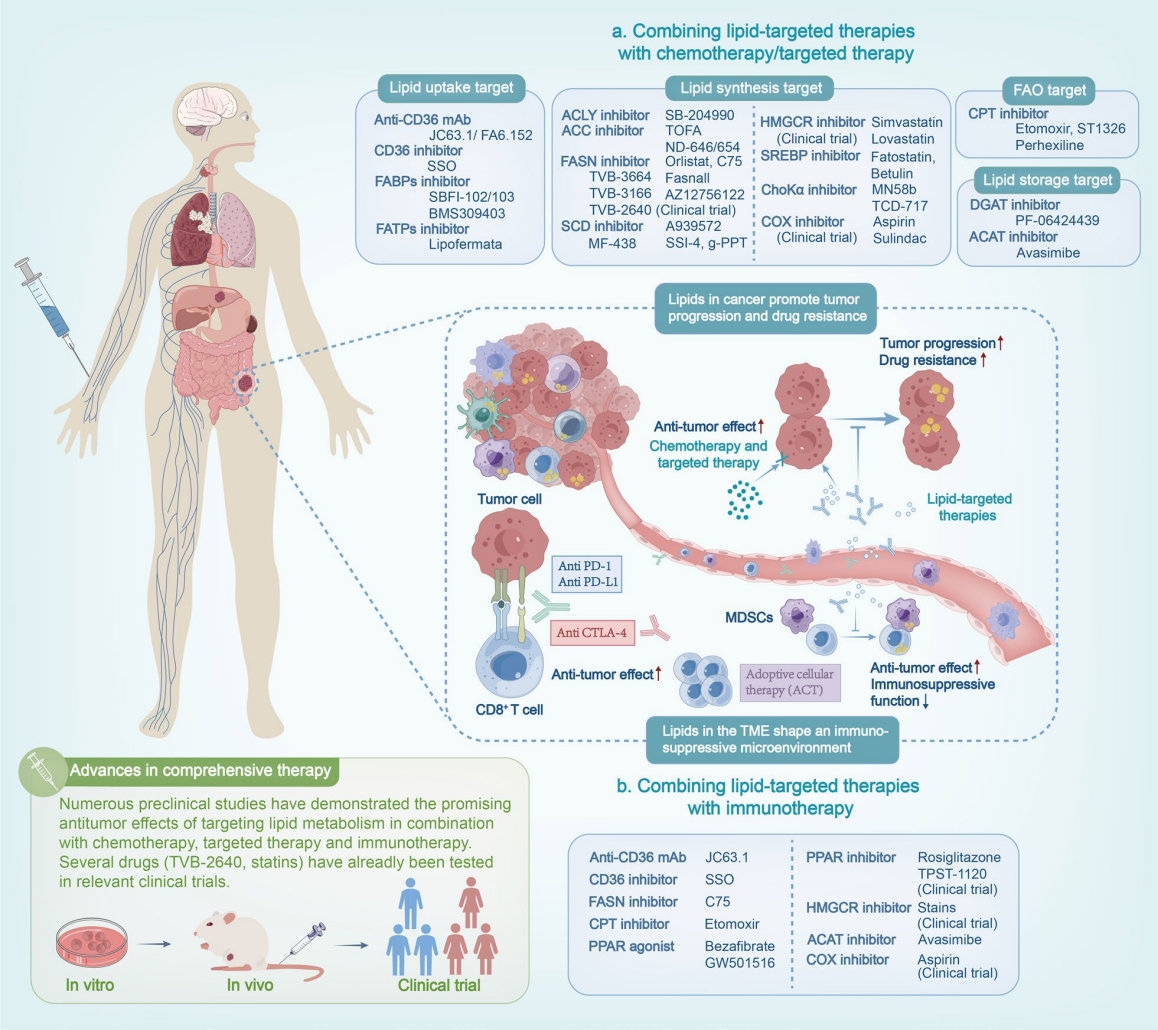
Lipid-targeted therapy in combination with chemotherapy, targeted therapy and immunotherapy. Lipid metabolism reprogramming within tumors not only propels tumor progression and drug resistance, but also play a crucial role in shaping the immunosuppressive microenvironment. This provides a theoretical foundation for the comprehensive treatment strategies by targeting tumor lipid metabolism. In numerous preclinical studies, inhibitors of lipid metabolism have shown significant anti-tumor effects in combination with chemotherapy, targeted therapy and immunotherapy, which is superior to a single-target treatment. The targets of these inhibitors primarily include lipid uptake (CD36, FABPs, FATPs), lipid synthesis (ACLY, ACCs, FASN, SCDs, SERBP, HMGCR, ChoK, COX), FAO (CPT1, PPAR), and lipid storage in LDs (DGAT, ACAT). These findings provide valuable insights for optimizing therapeutic strategy for cancer patients by combination lipid-targeted therapy with present anti-tumor therapy

Based on the comprehensive understanding of lipid metabolism in tumors and TME established in the previous text, targeting lipid metabolism holds promise as a multi-dimensional approach that can act on both tumor cells and TME cells, resulting in improved therapeutic outcomes. However, due to the plasticity of lipid metabolism, cancer cells can switch to an alternative pathway when one metabolic pathway is blocked. This, to some extent, hinders the anti-tumor efficacy of monotherapy [94, 223]. Notably, it can be effectively combined with tumor chemotherapy and targeted therapy to enhance anti-tumor efficacy in recent studies. Furthermore, targeting CD36 or CPT1 in combination with immunotherapy has shown promising results in enhancing anti-tumor immune responses. These findings provide valuable insights for optimizing therapeutic strategy for cancer patients (Fig. 5).

### Advances in targeting lipid metabolism combined with chemotherapy and targeted therapy

As described above, the dysregulated lipid metabolism in tumor cells provides energy support for tumor progression, playing an essential role in membrane synthesis and signal transduction. This shift in lipid metabolism, including the lipid uptake from the extracellular microenvironment, increased lipogenesis, and the increase in intracellular LD storage, correlates with the metastatic potential of tumor cells [224], the acquisition of stem cell-like properties [225], and the development of resistance to cancer chemotherapy [226, 227]. This may interfere with chemotherapy and targeted therapy in tumors. Therefore, aberrant lipid metabolism has become a potential target for treating drug-resistant cancer. Combination therapy can sensitize tumor cells to drugs, demonstrating a therapeutic effect superior to monotherapy [227]. Combination therapy also compensates for the insufficient efficacy of single-target lipid metabolism inhibitors. A considerable amount of preclinical research and clinical trials are currently focusing on novel strategies to enhance therapeutic effects by combining targeted lipid metabolism therapy with conventional chemotherapy and targeted therapy, even radiotherapy (Fig. 5a; Table 2).

**Table 2:** Combining lipid-targeted therapies with chemotherapy and targeted therapy

Target	Treatment	Phase	Tumor type	Combined treatment	Reference
CD36	JC63.1 (anti-CD36)	In vivo	Breast cancer	Lapatinib (HER2/EGFR inhibitor)	[231]
	FA6.152 (anti-CD36)	In vivo	Leukemia	Ara-C	[232]
	SSO (CD36 inhibitor)	In vivo	Leukemia	Ara-C, Doxorubicin, Etoposide, SN-38, Irinotecan, Dasatinib	[233]
FABPs	SBFI-102/103 (FABP5 inhibitor)	In vivo	Prostate cancer	Taxane	[236]
	BMS309403 (FABP4 inhibitor)	In vivo	Ovarian cancer	Carboplatin	[237]
FATPs	Lipofermata (FABP2 inhibitor)	In vivo	Melanoma	PLX4720 (BRAFi) PD0325901 (MEKi)	[238]
ACLY	SB-204990	In vivo	Melanoma	Vemurafenib (BRAFi)	[239]
		In vitro	Ovarian cancer	Cisplatin	[240]
ACC	TOFA	In vivo	HNSCC	Cetuximab	[241]
	ND-646	In vivo	NSCLC	Carboplatin	[242]
	ND-654	In vivo	HCC	Sorafenib	[243]
FASN	TVB-2640	Phase II	HER2^+^ Breast cancer	Taxane or trastuzumab	NCT03179904
		Phase II	Astrocytoma	Bevacizumab	NCT03032484 [258]
		Phase III	Glioblastoma	Bevacizumab	NCT05118776
		Phase I	NSCLC, ovarian, and breast cancer	Taxane	NCT02223247 [257]
	Orlistat	In vivo	Pancreatic cancer	Gemcitabine	[249]
		In vivo	Prostate cancer	Radiotherapy	[247]
		In vivo	Ovarian cancer	Cisplatin	[248]
		In vitro	HCC	Sorafenib	[245, 246]
		In vivo	NSCLC	Gefitinib (EGFRi)	[250]
	C75	In vivo	Breast cancer	Cetuximab	[319]
		In vivo	Gastrointestinal stromal tumors (GIST)	Imatinib	[320]
		In vivo	Breast cancer	CYH33 (PI3Ki)	[253]
	TVB-3664	In vivo	HCC	Cabozantinib, Sorafenib	[251]
		In vivo	Lung adenocarcinoma	MRTX849 (KRASi)	[252]
	TVB-3166	In vivo	Ovarian, prostate and pancreatic cancer	Taxane	[254]
	Fasnall	In vivo	HER2^+^ Breast cancer	Carboplatin	[255]
	AZ12756122	In vitro	NSCLC	Osimertinib	[256]
SCD	A939572	In vitro	Glioblastoma	Temozolomide	[262]
		In vivo	ccRCC	Temsirolimus (mTORi)	[261]
	SSI-4	In vivo	HCC	Sorafenib	[263]
	g-PPT	In vivo	NSCLC	Gefitinib	[264]
	MF-438	In vitro	Melanoma	Vemurafenib, Binimetinib	[260]
		In vitro	Lung cancer	Cisplatin	[265]
HMGCR	Simvastatin	Phase II	NSCLC	Gefitinib	NCT00452244 [275]
		Phase II	Breast cancer	Fluorouracil, adriamycin, and cyclophosphamide	NCT04418089 [276]
		Phase III	Prostate cancer	ADT	NCT03127631 [278]
		In vitro	Endometrial cancer	Metformin	[271]
		In vivo	Renal cell carcinoma	Everolimus	[270]
		In vitro	Glioblastoma	Temozolomide	[272]
	Lovastatin	In vivo	Gallbladder cancer	Cisplatin	[273]
		In vitro	Nasopharyngeal carcinoma	Cisplatin, cyclophosphamide, doxorubicin and paclitaxel	[274]
SREBP	Fatostatin	In vivo	Melanoma	Vemurafenib	[266]
		In vivo	Prostate cancer	Taxane	[321]
	Betulin	In vivo	HCC	Sorafenib	[267]
ChoK	MN58b TCD-717	In vivo In vitro	Colorectal cancer, PDAC	5-flurouracil	[279, 280]
COX	Aspirin	Phase II	ER^+^ breast cancer	ACT	NCT04038489
	Sulindac	Phase II	Colorectal cancer	Erlotinib	NCT01187901
CPT	Etomoxir	In vivo	Leukemia	Ara-C	[284]
		In vivo	Glioblastoma	Temozolomide	[322]
		In vivo	HCC	Antiangiogenic drug	[287]
		In vivo	Gastric cancer	5-fluorouracil	[285]
		In vivo	Nasopharyngeal carcinoma	Radiotherapy	[286]
		In vitro	Breast cancer	Radiotherapy	[323]
	Perhexiline	In vivo	Colorectal and gastric cancer	Oxaliplatin	[14]
		In vivo	PDAC	Gemcitabine	[289]
		In vivo	Ovarian cancer	Cisplatin	[290]
		In vitro	Breast cancer	Paclitaxel	[291]
	ST1326	In vivo	Leukemia	ABT199 (Bcl-2 inhibitor)	[324]
DGAT	PF-06424439	In vitro	Colorectal cancer	5-fluorouracil	[297]
		In vitro	Breast cancer	Cisplatin, radiotherapy	[298, 299]
ACAT	Avasimibe	In vivo	Leukemia	Imatinib	[325]
		In vivo	Breast cancer	Doxorubicin	[326]
		In vivo	Melanoma	Paclitaxel	[303]
		In vivo	PDAC	Gemcitabine	[304]
		In vitro	Biliary tract cancer	Gemcitabine	[305]

#### Targeting lipid uptake

Tumor cells exhibit a propensity for lipid uptake to support biosynthesis, energy production, and lipid storage in LDs. Consequently, inhibiting lipid uptake has emerged as a promising therapeutic strategy in oncology. Considerable preclinical evidence suggests that targeting the fatty acid receptor CD36 can be effective against various types of cancer [4, 15, 228, 229]. Particularly, CD36-mediated metabolic reprogramming in breast cancer, has been linked to resistance to HER2-targeted therapies. Recent clinical studies have confirmed that higher CD36 expression correlates with poorer outcomes in early-stage HER2^+^ breast cancer patients undergoing trastuzumab-lapatinib therapy [230]. Furthermore, JC63.1C, an anti-CD36 monoclonal antibody, was found to resensitize lapatinib-resistant xenograft tumors to HER2-targeted therapy [231], providing a novel direction for combinatorial treatment strategies in breast cancer. In addition, studies in leukemia models showed that the FA6.152 monoclonal anti-CD36 or the CD36 inhibitor SSO, when used in conjunction with chemotherapy drugs such as Ara-C or doxorubicin, led to a significant extension in survival [232, 233]. In PDAC, CD36 siRNA notably increased the efficacy of gemcitabine treatment [234].

Inhibiting FABPs has also demonstrated promising anti-tumor effects. FABP5 inhibitors, namely SBFI-102/103, were identified as novel targets in prostate cancer and were shown to enhance the tumor-suppressing effects of paclitaxel [235, 236]. FABP4 inhibitor BMS309403, was also found to boost the efficacy of carboplatin by inhibiting tumor metastasis in in vivo ovarian cancer models [237]. In another study, a mouse model of melanoma showed that inhibiting FATP2, another critical lipid metabolism transporter, in PMN-MDSCs using lipofermata, led to enhanced tumor regression when combined with BRAF/MEK inhibitor [238]. Taken together, these findings suggest that targeting lipid uptake by inhibiting fatty acid transporters is a promising new strategy to overcome therapy resistance in cancer treatment.

#### Targeting lipid synthesis

Increased lipid synthesis, inclusive of fatty acids and cholesterol, is a characteristic feature of tumors. Consequently, targeting key enzymes in these pathways is emerging as a potential therapeutic strategy for cancer, particularly in conjunction with chemotherapy and targeted therapy. ACLY, a key enzyme in the production of acetyl-CoA, serving as a substrate for DNL, supports tumor growth and confers resistance to vemurafenib in melanoma treatment. In tumor-bearing mice model, although vemurafenib exerted minimal effects on tumor growth, the combination of SB-204990 and vemurafenib exhibited a significantly more suppressive effect [239]. Similarly, in vitro results suggested that combining SB-204990 could be an effective strategy for treating cisplatin-resistant ovarian cancer [240]. In addition, ACCs, which catalyze the conversion of acetyl-CoA to malonyl-CoA, the initial step in fatty acid synthesis, are upregulated in cetuximab-treated head and neck squamous cell carcinoma (HNSCC) by the continuously activated AMPK pathway, suggesting a possible mechanism refers to the cetuximab resistance. A combination of cetuximab and TOFA, an ACC inhibitor, resulted in notable growth inhibition of cetuximab-resistant HNSCC xenografts in vivo [241]. Another ACC inhibitor, ND-646, when combined with carboplatin, could also significantly inhibit tumor growth in preclinical non-small cell lung cancer (NSCLC) models [242]. Similarly, the combination of sorafenib and a liver-specific ACC inhibitor (ND-654) effectively decreased tumor proliferation in vivo [243]. However, clinical trials assessing the efficacy of targeting ACC in tumors are currently scarce. Recent clinical trials have supported the use of an ACC inhibitor (Firsocostat) for treating nonalcoholic steatohepatitis (NCT02856555, NCT03987074) [244].

Palmitic acid synthesis, another rate-limiting step in DNL, is facilitated by FASN. Deregulation in the expression and activity of FASN carries significant implications for therapeutic response. FASN inhibitors, such as the anti-obesity drug Orlistat, have shown significant anti-tumor effects in various cancers [13]. Recent studies have underscored the efficacy of combining FASN inhibitors with other treatment strategies. Preclinical evidence suggests Orlistat's ability to reverse sorafenib resistance in HCC, thus illuminating new avenues for combined HCC treatment [245, 246]. Moreover, in prostate cancer, the combination of Orlistat and radiotherapy significantly reduces the activity of the NF-κB pathway, and inhibits cancer progression [247]. Preclinical research on pancreatic cancer, NSCLC, and ovarian cancer has shown that combining Orlistat with chemotherapy and targeted treatment significantly enhances anti-tumor effects [248–250]. Despite Orlistat being an FDA-approved therapy, there are currently no registered clinical trials examining its role in cancer. Another FASN inhibitor, TVB-3664, exhibits synergistic therapeutic effects when combined with TKI, as shown in an HCC mouse model [251]. A separate in vivo study using a lung adenocarcinoma xenograft model demonstrated the anti-tumor effects of combining TVB-3664 with a KRAS inhibitor [252]. The PI3K pathway, frequently hyperactive in tumor, plays crucial roles in both malignant and immune cells. The co-administration of the PI3K inhibitor (CYH33) with the FASN inhibitor (C75) has been found to synergistically inhibit tumor growth while enhancing host immunity [253]. Other FASN inhibitors like TVB-3166, Fasnall, and AZ12756122 also promote enhanced anti-tumor effects in combination therapies [254–256]. However, these inhibitors often present challenges related to poor pharmacokinetics, selectivity issues, and toxicity, rendering their clinical applicability uncertain. Notably, TVB-2640, considered the most promising FASN inhibitor, has been approved for multiple clinical studies.

When combined with paclitaxel, TVB-2640 has shown significant efficacy in clinical trials, including in patients with KRAS mutation in NSCLC, ovarian cancer and breast cancer, as compared to monotherapy (NCT02223247)[257]. In a phase II trial for relapsed high-grade astrocytoma, TVB-2640 was identified as a well-tolerated oral medication that could be safely administered in combination with bevacizumab. The positive safety profile and promising response rates endorse the initiation of a larger multicenter trial for TVB-2640 in conjunction with bevacizumab for astrocytoma (NCT03032484) [258]. Currently, a phase III clinical trial is underway to evaluate the combination of TVB-2640 and bevacizumab for the treatment of glioblastoma (NCT05118776). Furthermore, a phase II trial has been conducted to assess the efficacy of TVB-2640 in combination with trastuzumab and paclitaxel in patients with HER2^+^ metastatic breast cancer resistant to trastuzumab-based therapy (NCT03179904).

Targeting SCD1, the most critical enzyme in MUFA synthesis, has become the focus of many studies. SCD1 inhibitors, such as MF-438 and CAY10566, have been found to alter the composition of membrane PLs, leading to LPO and ferroptosis [259]. In vitro studies of BRAF-mutated melanoma have supported the role of MF-438 as a promising therapeutic target, especially when used in combination with MAPK inhibitors [260]. In ccRCC cells, the SCD1 inhibitor A939572 induces apoptosis and synergistically inhibits tumor growth when combined with temsirolimus, an mTOR inhibitor [261]. A range of SCD inhibitors is now available (e.g., A939572, SSI-4, g-PPT, MF-438), and most of these have been shown in preclinical models to produce stronger anti-tumor effects when combined with chemotherapy and targeted therapies [262–265]. However, few of these inhibitors have been translated into clinical trials, and none are currently being explored for their effects on cancer. SREBPs, key upstream regulators of lipid synthesis, are being considered as potential therapeutic targets, which can suppress the generation of key enzymes in lipid synthesis at the transcription level. Combining an SREBP inhibitor (fatostatin) with vemurafenib therapy enhances the therapy response in melanoma-bearing mice models, increasing membrane lipid polyunsaturation and lipid peroxidation [266]. Moreover, another preclinical study showed that the inhibition of SREBP-1 facilitated the anti-tumor effects of Sorafenib on HCC cells and xenograft tumors [267]. Importantly, a clinical trial focusing on head and neck cancer patients undergoing cisplatin chemotherapy demonstrated that dietary intake enriched with ω-3 FAs could prolong survival (NCT05101889) [268]. This also presents a potential novel dietary adjunctive therapeutic strategy.

Targeting cholesterol also provides a promising way to improve the sensitivity of anti-tumor drugs. Multiple studies have reported that the inhibition of cholesterol synthesis and uptake can notably impede proliferation and invasion in cancer. Statins, a type of HMGCR inhibitor, have effectively inhibited cholesterol in clinical trials [269]. A retrospective cohort study indicated that regular use of statins could lead to longer progression-free survival (PFS) in ccRCC patients prescribed with an mTOR inhibitor, everolimus, compared to those without statins. This finding has been confirmed in tumor xenograft models [270]. Mechanistically, the use of statins could represent a form of drug repositioning to enhance the efficacy of mTOR inhibitors. Similarly, statins combined with traditional chemotherapy have demonstrated promising results in preclinical models of endometrial, gallbladder cancer, nasopharyngeal carcinoma, and glioblastoma [271–274]. In an early-phase II trial of NSCLC, simvastatin showed potential to improve the efficacy of gefitinib in a subgroup of gefitinib-resistant NSCLC patients (NCT00452244) [275]. Moreover, a recent study on locally advanced breast cancer suggested that combining simvastatin with neoadjuvant chemotherapy improved therapeutic responses in patients. Although no statistically significant difference was documented, there was a trend toward better activity and tolerability (NCT04418089) [276]. However, a phase III trial in advanced gastric cancer reported no significant advantage of simvastatin plus capecitabine-cisplatin over placebo plus capecitabine-cisplatin (NCT01099085) [277]. The efficacy of statin combinations needs to be further validated in clinical trials for specific types of cancer. Currently, a phase III clinical trial is underway for prostate cancer patients, evaluating the combination of simvastatin with androgen deprivation therapy (ADT), with promising results anticipated (NCT03127631) [278].

PL metabolism and lipid-derived mediators (e.g., PGE2 and LPA) are crucial in tumor progression and drug resistance, suggesting new strategies for comprehensive therapy. ChoK, the key enzyme for PCho phosphorylation, is overexpressed in various tumors. ChoKα inhibitors, MN58b and TCD-717, have shown effective anti-tumor activity combined with 5-fluorouracil in mouse xenograft models of colorectal cancer [279]. Similarly, MN58b synergizes with gemcitabine, 5-fluorouracil and oxaliplatin in preclinical studies of PDAC [280]. TCD-717 has entered Phase I trials as a novel anti-tumor strategy, but no data are available yet (NCT01215864). Furthermore, other key enzymes in PL metabolism, including PLC and D, have been demonstrated as potential anti-tumor targets in some preclinical studies. D609, a PLC inhibitor, can induce proliferation arrest and cell differentiation in breast cancer cells [281]. Similarly, targeted inhibition of PLD effectively suppresses tumor growth and metastasis in mouse model [282]. Notably, inhibiting bioactive lipids, especially through PGE2 pathway by using COX inhibitors, has shown efficacy in various tumors, including gastrointestinal cancer [283]. Several clinical trials have evaluated the therapeutic potential of COX inhibitors in combination therapies, highlighting their significance in treatment regimen (NCT04038489) (NCT01187901).

#### Targeting FAO

FAO, which involves the key rate-limiting enzyme CPT, has emerged as a promising metabolic therapeutic target for cancer. Etomoxir, the most commonly used CPT inhibitor in preclinical research, has shown notable efficacy when combined with chemotherapy and targeted treatments, functioning through the inhibition of mitochondrial FAO. For example, blocking CPT1 with etomoxir significantly enhances the cytotoxicity of Ara-C against drug-resistant leukemia cells [284]. Likewise, numerous in vitro preclinical studies have indicated a promising future for etomoxir when combined with traditional chemotherapy and radiotherapy [285, 286]. Moreover, antiangiogenic drug (AAD)-induced tumor hypoxia initiates FAO reprogramming, which stimulates cancer cell proliferation in HCC therapy. The combination of AAD and the FAO inhibitor etomoxir can resensitize drug-resistant tumor cells and enhance anti-tumor effects [287]. However, few clinical studies have used etomoxir for anti-cancer treatment due to its clear hepatotoxicity [288]. Perhexiline, another CPT inhibitor, when combined with oxaliplatin, significantly suppressed the progression of gastrointestinal cancer in cell-based xenograft and patient-derived xenograft models. In preclinical models of PDAC, ovarian, and breast cancer, perhexiline combined with chemotherapy has demonstrated potential application value [289–291]. Notably, perhexiline has been approved for angina treatment in Australia and Asia, therefore it could be translated quite safely into anti-tumor trials. While clinical trials for anti-tumor treatment have not yet been conducted with CPT inhibitors like etomoxir, perhexiline, and ST1326, substantial preclinical evidence provides promising therapeutic strategies [14, 292].

#### Targeting lipid storage

LDs are storage organelles characterized by a lipid core containing neutral lipids. They play a fundamental role in tumor cells and are associated with tumor aggressiveness and therapy resistance [293–295]. For example, LD accumulation drives cell death resistance to 5-fluorouracil and oxaliplatin treatments both in vitro and in vivo. Mechanistically, LD accumulation impairs caspase cascade activation and ER stress responses [295]. Inhibition of LD synthesis has been shown to upregulate LPO, indicating its potential as a cancer therapy. Numerous studies have linked DGAT to the progression of several cancers, such as gastric cancer, which is dependent on promoting lipid storage in LDs [107, 129, 296]. The DGAT inhibitor, PF-06424439, has been confirmed in vitro studies of colorectal and breast cancer to have a positive role in enhancing the anti-tumor effects of 5-fluorouracil, cisplatin, and even radiotherapy [297–299]. Additionally, inhibition of CE formation has also been identified as a promising approach to suppress tumor proliferation and metastasis. Avasimibe, an ACAT-1 inhibitor, elevates the level of free cholesterol in pancreatic, colorectal, and liver cancer cells, inducing ER stress and resulting in cancer cell apoptosis [300–302]. Furthermore, avasimibe was combined with chemotherapy (paclitaxel and the immunoadjuvant αGC), leading to enhanced CTL responses by facilitating the formation of T cell receptors and anti-tumor effects in B16 melanoma xenograft models [303]. The combination of avasimibe and gemcitabine demonstrated a strong synergistic effect in suppressing PDAC and biliary tract cancer in vitro and tumor growth in vivo [304, 305]. However, to date, no inhibitors designed to target DGAT and ACAT have been tested in clinical trials in cancer patients.

### Advances in targeting lipid metabolism combined with immunotherapy

Cancer immunotherapies, including checkpoint inhibitors and adoptive cell transfer (ACT) [306], which activate the immune system to recognize and attack cancer cells, have obtained durable clinical responses. Immune checkpoint inhibitors (ICIs) are monoclonal antibodies and comprise anti-programmed cell death protein 1 (PD-1), anti-cytotoxic T-lymphocyte-associated protein 4 (CTLA-4), and anti-programmed death-ligand 1 (PD-L1), which targets immune inhibitory pathways known as checkpoints. It is important to note that ICIs are not believed to kill tumor cells directly. Rather, they mediate their anti-tumor effects indirectly by stimulating T cells and enhancing their functions, making ICIs more effective and less toxic than traditional systemic immune therapies [307, 308]. Current studies increasingly focus on the role of lipid metabolic reprogramming in immune cells on immunotherapy. Building on the previously mentioned understanding, it has been noted that immune cells within the TME amplify their immunosuppressive effects by altering their lipid metabolism patterns, which subsequently promotes tumor progression. As an illustration, disrupting PD-L1 palmitoylation makes cancer cells more susceptible to T cell-mediated destruction, thereby inhibiting tumor growth [309, 310]. This suggests a theoretical foundation for modifying the immunosuppressive TME through the targeting of lipid metabolism. Moreover, several metabolism-modulating drugs such as statins and bezafibrate have been shown to obstruct cancer development [164, 311], indicating a novel therapeutic strategy that combines metabolism-regulating agents with immunotherapy for the treatment of cancer. Thus, we aim to summarize promising therapeutic targets within various lipid metabolism pathways including lipid uptake, synthesis, and FAO, in relation to immunotherapy (Fig. 5b; Table 3).

**Table 3:** Combining lipid-targeted therapies with immunotherapy

Target	Treatment	Phase	Tumor type	Effects on immune cells	Combined treatment	Reference
CD36	JC63.1 (Anti-CD36)	In vivo	B16 melanoma lung metastatic model	Reduced ferroptosis and enhanced anti-tumor function in CD8^+^ T cells	Anti-PD-1 Ab	[169]
		In vivo	YUMM1.7 melanoma-bearing mice model	Reduced accumulation and promoted apoptosis in Treg cells Increased accumulation in CD8^+^ TILs	Anti-PD-1 Ab	[8]
	SSO	In vivo	HCC murine models	Decreased Tregs and MDSCs Increased IFN-γ^+^ and granzyme B^+^ CD8^+^ T cells	Anti-PD-1 Ab	[153]
FATP2	Lipofermata	In vivo	TC-1 and LLC tumor-bearing mice model	Promoted anti-tumor effect of CD8^+^ TILs	Anti-CTLA4 Ab	[10]
		In vivo	B16 melanoma and LLC tumor-bearing mice model	Activated T cells and inhibited suppressive role of MDSCs	Anti-PD-L1 Ab	[221]
FASN	C75	In vitro	TAMs coculture with TPC-1 thyroid tumor	Reduced extracellular cytokine levels from TAMs through inhibition of lipid biosynthesis	NA	[191]
CPT	Etomoxir	In vitro	TAMs coculture with various tumor cell lines	Suppressed pro-tumor function of TAMs through inhibition of FAO	NA	[187]
		In vivo	Lewis lung and MC38 colon tumor-bearing mice	Increased number of adoptively transferred OT-1 T cells infiltrating the tumors and cells producing IFN-γ	ACT	[222]
		In vivo	GL261 glioblastoma-bearing mice model	Boost TAM phagocytosis and anti-tumor effect	Anti-CD47 Ab	[312]
PPAR	Bezafibrate (PGC-1α/PPAR agonist)	In vivo	MC38 colon tumor-bearing mice	Enhanced proliferation during the early phase and inhibited apoptosis of the effector T cells	Anti-PD-1 Ab	[164]
		In vivo	Lewis lung tumor-bearing mice	Maintained survival and functional capacity of CD8^+^ TILs	Anti-PD-L1 Ab	[313]
	GW501516 (PPAR agonist)	In vivo	B16 melanoma-bearing mice	Activated the expression of T-bet and IFN-γ level in CD8^+^ T cells	ACT	[314]
	Rosiglitazone (PPARγ inhibitor)	Phase II	Solid Tumor Malignancies	NA	Nivolumab, Pembrolizumab	NCT04114136
	TPST-1120 (PPARα inhibitor)	Phase I	Advanced Cancers	NA	Nivolumab	NCT03829436
HMGCR	Statin	In vivo	MOC1 oral tumor-bearing mice	Activated effector T cells Shifted macrophages from M2 to M1	Anti-PD-1 Ab	[315]
		Prospective cohort	Pleural mesothelioma, NSCLC	NA	Nivolumab	[311]
		Retrospective study	NSCLC	NA	Nivolumab, Pembrolizumab	[316]
		Prospective cohort	NSCLC	NA	PD-1/PD-L1 inhibitors	NCT05636592
PSK9	Evolocumab	In vivo	MC38 colon tumor-bearing mice	Boost the number of active IFN-γ^+^ CTLs	Anti-PD-1 Ab	[317]
ACAT	Avasimibe	In vivo	B16 melanoma and lung tumor-bearing mice	Enhanced anti-tumor effect and cytokine production of CD8^+^ T cells	Anti-PD-L1 Ab	[172]
		In vivo	B16 melanoma-bearing mice	Increased tumor cell apoptosis and T cell effect	ACT	[303]
		Tissue	HCC	Enhanced expansion of cytolytic and non-cytolytic antigen-specific CD8^+^ T cells	Nivolumab	[318]
COX	Asprin	Phase II	MSI-H colorectal cancer	NA	Anti-PD-1 Ab	NCT03638297

As a critical regulator of lipid metabolism in immune cells, CD36 significantly influences their anti-tumor immune responses within the TME. Suppressing CD36 in CD8^+^ T cells reduces intracellular lipid accumulation and reinstates their anti-tumor activity [169], while targeting CD36 in Treg cells mitigates their immunosuppressive effects [8]. Combined, these strategies can synergistically augment the efficacy of anti-PD-1 therapy. The potency of this conclusion has been substantiated through the conjunction of JC63.1 and anti-PD-1 mAb in the melanoma-bearing mice model. Similarly, CD36 inhibitor SSO synergizes with anti-PD-1 immunotherapy by restoring anti-tumor T cell responses in HCC. This combination treatment exhibits marked anti-tumor efficacy with decreased Tregs and MDSCs and increased IFN-γ^+^ and granzyme B^+^ CD8^+^ T cells [153]. In addition, directing the intervention toward CD36 in TAMs can also decrease their internal lipid accumulation and affect their functional phenotypes [187]. In gastric cancer, PD-L1 blockade augments the expression of FABP4/5 and lipid uptake of tissue-resident memory T cells (Trm), thereby extending their lifespan and enhancing their anti-tumor effects [168]. Furthermore, FATP2 is a vital regulator of the immunosuppressive function of PMN-MDSCs, which mediates its effect via regulation of lipid accumulation and subsequent synthesis of PGE2. A combination treatment involving lipofermata and a CTLA4 antibody in TC-1 and LLC tumor-bearing mice demonstrated a synergistic tumor-suppressive effect compared to treatment with either agent alone. Mechanistically, the anti-tumor effect was attributed to a significant infiltration of CD8^+^ T cells within the tumors, achieved by inhibiting PMN-MDSCs [10]. The inhibition of FATP2 expression in MDSCs, in combination with anti-PD-L1 therapy, can also significantly enhance the anti-tumor effect through the regulation of ROS in MDSCs [221]. Therefore, targeting lipid uptake presents an effective approach to potentiate cancer immunotherapy, which has demonstrated superior anti-tumor efficacy in mouse models.

Apart from lipid uptake, FAS and FAO also play critical roles in tumor-associated immune cells and serve as potential targets for combination immunotherapy. Tumor-infiltrating Treg cells display heightened PD-1 expression, which is dependent on the activation of SREBP, revealing a potential strategy for targeting Treg cell lipid metabolism for cancer immunotherapy [176]. An intervention known as C57, which targets FASN in TAMs, can reduce its secretion of pro-tumor inflammatory factors and exert a tumor-suppressive effect [191]. Additionally, CYH33, in combination with C75, induces immune activation and enhances anti-tumor immunity, providing a rationale for the concurrent targeting of PI3K and FASN in breast cancer treatment [253]. A combination of the CPT1 inhibitor etomoxir with ACT suppresses the immunosuppressive function of tumor-infiltrating MDSCs, leading to significant anti-tumor effects [222]. In the TME, etomoxir can inhibit the FAO of TAMs, reduce CD47-mediated anti-phagocytosis, and promote anti-tumor immunity in glioblastoma and hematological tumors [187, 312]. Additionally, a preclinical study involving MC38 colon tumor-bearing mice demonstrated that the PPARγ coactivator 1-α (PGC-1α)/PPAR agonist, bezafibrate, could elevate the levels of CPT1, enhance FAO, and preserve the population of CD8^+^ TILs in TME. This mechanism thus aids in the facilitation of anti-PD-L1 therapy [164]. In lung cancer models, bezafibrate further amplified the effects of PD-L1 blockade by fostering the expansion of effector T cells within the TME [313]. Similarly, GW501516, another PPAR agonist, improved the potency of ACT by modifying T cell metabolism and cytokine expression in B16 melanoma-bearing mouse models [314]. Strikingly, current clinical trials are evaluating the efficacy of combining PD-1 blockade with PPARs inhibitors, such as rosiglitazone and TPST-1120 (NCT03829436, NCT04114136), in various cancer. The latter study primarily focuses on the proliferation of T cells and the enhancement of anti-tumor immunity. In summary, the strategic targeting of FAO synthesis and oxidation, along with immunotherapy, represents a promising approach for precision therapy.

Currently, cholesterol is a subject of keen interest in the field of tumor immunotherapy. Particularly, inhibitors of HMGCR, such as statins, have demonstrated potential as therapeutic targets. Studies conducted in mice models have shown that daily oral administration of simvastatin or lovastatin enhances tumor control and prolongs survival when combined with PD-1 blockade. These findings suggest T cell activation and the transition from M2 to M1 macrophage predominance as possible mechanisms underlying combination therapy [315]. Furthermore, statins have exhibited potential to enhance the effects of immunotherapy in two clinical studies conducted on NSCLC and pleural mesothelioma patients [311, 316]. Currently, a prospective clinical study (NCT05636592) is underway to investigate the treatment of NSCLC using a combination of statins and PD-1/PD-L1 inhibitors. Moreover, inhibiting PCSK9, a key protein in the regulation of cholesterol metabolism, can boost the response of tumors to immune checkpoint therapy [317]. Avasimibe inhibiting ACAT can block cholesterol ester synthesis and LD formation, then enhance the anti-tumor effect of CD8^+^ TILs by increasing membrane synthesis and intracellular free cholesterol levels [172]. Furthermore, ACAT inhibition can enhance the in vitro sensitivity of CD8^+^ T cells to PD-1 blockade in HBV-related HCC [318]. In a preclinical study conducted on MC38 colon tumor-bearing mice, the combination of avasimibe and an anti-PD-1 antibody displayed superior efficacy in controlling tumor progression compared to either monotherapy, resulting in a significant anti-tumor effect [172]. Notably, a recent Phase II clinical trial was conducted to investigate the efficacy of a COX inhibitor combined with PD-1 antibody in treating MSI-H colorectal cancer (NCT03638297). There is substantial preclinical evidence supporting the feasibility of targeting lipid metabolism in conjunction with immunotherapy. Anticipation is building for future clinical trials to further validate and translate these potential targets into novel treatment strategies for cancer patients.

## Conclusions and perspectives

Lipid play a critical role in cancer progression by serving as energy sources, membrane structures, and signaling molecules. Tumor cells often undergo lipid metabolic reprogramming, characterized by increased lipid uptake, lipid synthesis, fatty acid oxidation, and lipid storage, to survive and develop under hypoxic and nutrient-deficient conditions. Gene mutations and environmental factors (hypoxia, acidosis, and nutritional deficiency) are important drivers to promote metabolic reprogramming in tumor cells. Metabolic reprogramming of tumor cells will further remodel the TME, affecting lipid metabolism and functional phenotypes of TME cells. Among them, lipid metabolic reprogramming of CAFs, Treg cells, CD8^+^ T cells, and TAMs plays an important role in shaping the tumor immunosuppressive microenvironment, leading to tumor immune escape and therapeutic resistance, ultimately promoting tumor progression. Based on the above facts, targeting lipid metabolism holds a promising multi-dimensional therapeutic approach for cancer. However, drugs targeting metabolic pathways are currently in the preclinical stage, even though there is strong evidence that combining metabolic inhibitors with chemotherapy or immunotherapy can significantly enhance their anti-tumor efficacy in animal models. Given the complex connections between different metabolites, the development of useful drugs targeting tumor metabolic pathways still has a long way to go.

## Data Availability

Not applicable.

## Abbreviations

FAO: Fatty acid oxidation
TME: Tumor microenvironment
CAFs: Cancer-associated fibroblasts
Tregs: Regulatory T cells
TAMs: Tumor-associated macrophages
MDSCs: Myeloid-derived suppressor cells
NK cells: Natural killer cells
TAG: Triacylglycerols
FA: Fatty acid
DNL: De novo lipogenesis
PL: Phospholipid
FATP: Fatty acid transport protein
FABP: Fatty acid-binding protein
ACLY: ATP-citrate lyase
ACSS: Acetyl-CoA synthetase
ACC: Acetyl-CoA carboxylases
FASN: Fatty acid synthase
SFA: Saturated fatty acid
MUFA: Monounsaturated fatty acid
PUFA: Polyunsaturated fatty acid
ELOVLs: Elongation of very-long-chain fatty acids gene family
SCD: Stearoyl-CoA desaturase
FADS: Fatty acid desaturase
OA: Oleic acid
LA: Linoleic acid
ALA: Alpha-linolenic acid
AA: Arachidonic acid
AdA: Adrenic acid
GLS: Glutaminase
GLUD: Glutamate dehydrogenase
IDH: Isocitrate dehydrogenase
LCFA: Long-chain fatty acid
GPAT: Glycerol-3-phosphate acyltransferase
LPA: Lysophosphatidic acid
DAG: Diacylglycerol
DGAT: Diacylglycerol acyltransferase
MVA: Mevalonate
HMGCR: HMG-CoA reductase
ABCA1: ATP-binding cassette transporter A1
LDLR: Low-density lipoprotein receptor
PC: Phosphatidylcholine
PCho: Phosphocholine
GPC: Glycerophosphocholine
ChoK: Choline kinase
PA: Phosphatidic acid
LPC: Lysophosphatidylcholine
LPCATs: Lysophosphatidylcholine acyltransferase
ATX: Autotaxin
PG: Prostaglandin
COX: Cyclooxygenase
LT: Leukotriene
LOX: Lipoxygenase
S1P: Sphingosine-1-phosphate
ACAT: Acyl-CoA cholesterol acyltransferase
ER: Endoplasmic reticulum
LD: Lipid droplet
ATGL: Adipose triglyceride lipase
HSL: Hormone-sensitive lipase
MAL: Monoacylglycerol lipase
HIG2: Hypoxia-inducible gene 2
CPT: Carnitine palmitoyl transferase
ACSL: The long-chain acyl-CoA synthase enzyme family
LPO: Lipid peroxidation
PUFA-PL: PUFA-phospholipid
MUFA-PL: MUFA-phospholipid
SREBP: Sterol regulatory element-binding protein
RTK: Receptor tyrosine kinase
PPP: Pentose phosphate pathway
HCC: Hepatocellular carcinoma
HIF: Hypoxia-inducible factor
ccRCC: Clear cell renal cell carcinoma
LRP1: Low-density lipoprotein receptor-related protein 1
FAS: Fatty acid synthesis
PPAR: Peroxisome proliferator activated receptor
HFD: High-fat diet
EVs: Extracellular vesicles
ALOX15: Arachidonate lipoxygenase 15
CD8^+^ TILs: CD8^+^ tumor-infiltrating lymphocytes
ox-LDL: Oxidized low-density lipoprotein
PDAC: Pancreatic ductal adenocarcinoma
GPX4: Glutathione peroxidase 4
MARCO: Macrophage receptor for collagenous structures
ICI: Immune checkpoint inhibitor
M-MDSC: Mononuclear MDSC
PMN-MDSC: Polymorphonuclear MDSC
NSCLC: Non-small cell lung cancer
HNSCC: Neck squamous cell carcinoma
ADT: Androgen deprivation therapy
AAD: Antiangiogenic drug
ACT: Adoptive cell transfer
PD-1: Programmed cell death protein 1
CTLA-4: Cytotoxic T-lymphocyte-associated protein 4
PD-L1: Programmed death-ligand 1
PGC-1α: PPARγ coactivator 1-α

## References

[1] Hanahan D, Weinberg RA (2011). Hallmarks of cancer: the next generation. Cell.

[2] Rohrig F, Schulze A (2016). The multifaceted roles of fatty acid synthesis in cancer. Nat Rev Cancer.

[3] Shang S, Liu J, Hua F (2022). Protein acylation: mechanisms, biological functions and therapeutic targets. Signal Transduct Target Ther.

[4] Koundouros N, Poulogiannis G (2020). Reprogramming of fatty acid metabolism in cancer. Br J Cancer.

[5] Strickaert A, Saiselet M, Dom G, De Deken X, Dumont JE, Feron O, Sonveaux P, Maenhaut C (2017). Cancer heterogeneity is not compatible with one unique cancer cell metabolic map. Oncogene.

[6] Liu Y, Cao X (2016). Characteristics and significance of the pre-metastatic niche. Cancer Cell.

[7] Bader JE, Voss K, Rathmell JC (2020). Targeting metabolism to improve the tumor microenvironment for cancer immunotherapy. Mol Cell.

[8] Wang H, Franco F, Tsui YC, Xie X, Trefny MP, Zappasodi R, Mohmood SR, Fernandez-Garcia J, Tsai CH, Schulze I (2020). CD36-mediated metabolic adaptation supports regulatory T cell survival and function in tumors. Nat Immunol.

[9] Wu L, Zhang X, Zheng L, Zhao H, Yan G, Zhang Q, Zhou Y, Lei J, Zhang J, Wang J (2020). RIPK3 orchestrates fatty acid metabolism in tumor-associated macrophages and hepatocarcinogenesis. Cancer Immunol Res.

[10] Veglia F, Tyurin VA, Blasi M, De Leo A, Kossenkov AV, Donthireddy L, To TKJ, Schug Z, Basu S, Wang F (2019). Fatty acid transport protein 2 reprograms neutrophils in cancer. Nature.

[11] Xu S, Chaudhary O, Rodriguez-Morales P, Sun X, Chen D, Zappasodi R, Xu Z, Pinto AFM, Williams A, Schulze I (2021). Uptake of oxidized lipids by the scavenger receptor CD36 promotes lipid peroxidation and dysfunction in CD8(+) T cells in tumors. Immunity.

[12] Niavarani SR, Lawson C, Bakos O, Boudaud M, Batenchuk C, Rouleau S, Tai LH (2019). Lipid accumulation impairs natural killer cell cytotoxicity and tumor control in the postoperative period. BMC Cancer.

[13] Schcolnik-Cabrera A, Chavez-Blanco A, Dominguez-Gomez G, Taja-Chayeb L, Morales-Barcenas R, Trejo-Becerril C, Perez-Cardenas E, Gonzalez-Fierro A, Duenas-Gonzalez A (2018). Orlistat as a FASN inhibitor and multitargeted agent for cancer therapy. Expert Opin Investig Drugs.

[14] Wang Y, Lu JH, Wang F, Wang YN, He MM, Wu QN, Lu YX, Yu HE, Chen ZH, Zhao Q (2020). Inhibition of fatty acid catabolism augments the efficacy of oxaliplatin-based chemotherapy in gastrointestinal cancers. Cancer Lett.

[15] Ruan C, Meng Y, Song H (2022). CD36: an emerging therapeutic target for cancer and its molecular mechanisms. J Cancer Res Clin Oncol.

[16] Zhang C, Liao Y, Liu P, Du Q, Liang Y, Ooi S, Qin S, He S, Yao S, Wang W (2020). FABP5 promotes lymph node metastasis in cervical cancer by reprogramming fatty acid metabolism. Theranostics.

[17] Bensaad K, Favaro E, Lewis CA, Peck B, Lord S, Collins JM, Pinnick KE, Wigfield S, Buffa FM, Li JL (2014). Fatty acid uptake and lipid storage induced by HIF-1alpha contribute to cell growth and survival after hypoxia-reoxygenation. Cell Rep.

[18] Zhang M, Di Martino JS, Bowman RL, Campbell NR, Baksh SC, Simon-Vermot T, Kim IS, Haldeman P, Mondal C, Yong-Gonzales V (2018). Adipocyte-derived lipids mediate melanoma progression via FATP proteins. Cancer Discov.

[19] Mendes C, Lopes-Coelho F, Ramos C, Martins F, Santos I, Rodrigues A, Silva F, Andre S, Serpa J (2019). Unraveling FATP1, regulated by ER-beta, as a targeted breast cancer innovative therapy. Sci Rep.

[20] Wang D, Ye Q, Gu H, Chen Z (2022). The role of lipid metabolism in tumor immune microenvironment and potential therapeutic strategies. Front Oncol.

[21] Snaebjornsson MT, Janaki-Raman S, Schulze A (2020). Greasing the wheels of the cancer machine: the role of lipid metabolism in cancer. Cell Metab.

[22] Khwairakpam AD, Banik K, Girisa S, Shabnam B, Shakibaei M, Fan L, Arfuso F, Monisha J, Wang H, Mao X (2020). The vital role of ATP citrate lyase in chronic diseases. J Mol Med (Berl).

[23] Comerford SA, Huang Z, Du X, Wang Y, Cai L, Witkiewicz AK, Walters H, Tantawy MN, Fu A, Manning HC (2014). Acetate dependence of tumors. Cell.

[24] Jones SF, Infante JR (2015). Molecular pathways: fatty acid synthase. Clin Cancer Res.

[25] Ferraro GB, Ali A, Luengo A, Kodack DP, Deik A, Abbott KL, Bezwada D, Blanc L, Prideaux B, Jin X (2021). Fatty acid synthesis is required for breast cancer brain metastasis. Nat Cancer.

[26] Zhao J, Zhi Z, Wang C, Xing H, Song G, Yu X, Zhu Y, Wang X, Zhang X, Di Y (2017). Exogenous lipids promote the growth of breast cancer cells via CD36. Oncol Rep.

[27] Ascenzi F, De Vitis C, Maugeri-Sacca M, Napoli C, Ciliberto G, Mancini R (2021). SCD1, autophagy and cancer: implications for therapy. J Exp Clin Cancer Res.

[28] Cluntun AA, Lukey MJ, Cerione RA, Locasale JW (2017). Glutamine metabolism in cancer: understanding the heterogeneity. Trends Cancer.

[29] Jin J, Byun JK, Choi YK, Park KG (2023). Targeting glutamine metabolism as a therapeutic strategy for cancer. Exp Mol Med.

[30] Hui S, Ghergurovich JM, Morscher RJ, Jang C, Teng X, Lu W, Esparza LA, Reya T, Le Z, Yanxiang Guo J (2017). Glucose feeds the TCA cycle via circulating lactate. Nature.

[31] Ippolito L, Comito G, Parri M, Iozzo M, Duatti A, Virgilio F, Lorito N, Bacci M, Pardella E, Sandrini G (2022). Lactate rewires lipid metabolism and sustains a metabolic-epigenetic axis in prostate cancer. Cancer Res.

[32] Perez-Escuredo J, Dadhich RK, Dhup S, Cacace A, Van Hee VF, De Saedeleer CJ, Sboarina M, Rodriguez F, Fontenille MJ, Brisson L (2016). Lactate promotes glutamine uptake and metabolism in oxidative cancer cells. Cell Cycle.

[33] Broadfield LA, Pane AA, Talebi A, Swinnen JV, Fendt SM (2021). Lipid metabolism in cancer: new perspectives and emerging mechanisms. Dev Cell.

[34] Butler LM, Perone Y, Dehairs J, Lupien LE, de Laat V, Talebi A, Loda M, Kinlaw WB, Swinnen JV (2020). Lipids and cancer: emerging roles in pathogenesis, diagnosis and therapeutic intervention. Adv Drug Deliv Rev.

[35] Kuzu OF, Noory MA, Robertson GP (2016). The role of cholesterol in cancer. Cancer Res.

[36] Gallagher EJ, Zelenko Z, Neel BA, Antoniou IM, Rajan L, Kase N, LeRoith D (2017). Elevated tumor LDLR expression accelerates LDL cholesterol-mediated breast cancer growth in mouse models of hyperlipidemia. Oncogene.

[37] Kim HY, Bae SJ, Choi JW, Han S, Bae SH, Cheong JH, Jang H (2022). Cholesterol synthesis is important for breast cancer cell tumor sphere formation and invasion. Biomedicines.

[38] Halimi H, Farjadian S (2022). Cholesterol: an important actor on the cancer immune scene. Front Immunol.

[39] Kopecka J, Trouillas P, Gasparovic AC, Gazzano E, Assaraf YG, Riganti C (2020). Phospholipids and cholesterol: inducers of cancer multidrug resistance and therapeutic targets. Drug Resist Updat.

[40] Glunde K, Bhujwalla ZM, Ronen SM (2011). Choline metabolism in malignant transformation. Nat Rev Cancer.

[41] Saito RF, Andrade LNS, Bustos SO, Chammas R (2022). Phosphatidylcholine-derived lipid mediators: the crosstalk between cancer cells and immune cells. Front Immunol.

[42] Iorio E, Ricci A, Bagnoli M, Pisanu ME, Castellano G, Di Vito M, Venturini E, Glunde K, Bhujwalla ZM, Mezzanzanica D (2010). Activation of phosphatidylcholine cycle enzymes in human epithelial ovarian cancer cells. Cancer Res.

[43] Hernando E, Sarmentero-Estrada J, Koppie T, Belda-Iniesta C, Ramirez de Molina V, Cejas P, Ozu C, Le C, Sanchez JJ, Gonzalez-Baron M (2009). A critical role for choline kinase-alpha in the aggressiveness of bladder carcinomas. Oncogene.

[44] Sulciner ML, Gartung A, Gilligan MM, Serhan CN, Panigrahy D (2018). Targeting lipid mediators in cancer biology. Cancer Metastasis Rev.

[45] Shindou H, Hishikawa D, Harayama T, Yuki K, Shimizu T (2009). Recent progress on acyl CoA: lysophospholipid acyltransferase research. J Lipid Res.

[46] Hisano Y, Hla T (2019). Bioactive lysolipids in cancer and angiogenesis. Pharmacol Ther.

[47] Li Z, Liu H, Luo X (2020). Lipid droplet and its implication in cancer progression. Am J Cancer Res.

[48] Petan T (2023). Lipid droplets in cancer. Rev Physiol Biochem Pharmacol.

[49] Moldavski O, Amen T, Levin-Zaidman S, Eisenstein M, Rogachev I, Brandis A, Kaganovich D, Schuldiner M (2015). Lipid droplets are essential for efficient clearance of cytosolic inclusion bodies. Dev Cell.

[50] Cheng X, Geng F, Pan M, Wu X, Zhong Y, Wang C, Tian Z, Cheng C, Zhang R, Puduvalli V (2020). Targeting DGAT1 ameliorates glioblastoma by increasing fat catabolism and oxidative stress. Cell Metab.

[51] Wilcock DJ, Badrock AP, Wong CW, Owen R, Guerin M, Southam AD, Johnston H, Telfer BA, Fullwood P, Watson J (2022). Oxidative stress from DGAT1 oncoprotein inhibition in melanoma suppresses tumor growth when ROS defenses are also breached. Cell Rep.

[52] Zhang R, Meng J, Yang S, Liu W, Shi L, Zeng J, Chang J, Liang B, Liu N, Xing D (2022). Recent advances on the role of ATGL in cancer. Front Oncol.

[53] Rossi T, Zamponi R, Chirico M, Pisanu ME, Iorio E, Torricelli F, Gugnoni M, Ciarrocchi A, Pistoni M (2023). BETi enhance ATGL expression and its lipase activity to exert their antitumoral effects in triple-negative breast cancer (TNBC) cells. J Exp Clin Cancer Res.

[54] Zhang X, Saarinen AM, Hitosugi T, Wang Z, Wang L, Ho TH, Liu J (2017). Inhibition of intracellular lipolysis promotes human cancer cell adaptation to hypoxia. Elife.

[55] Povero D, Johnson SM, Liu J (2020). Hypoxia, hypoxia-inducible gene 2 (HIG2)/HILPDA, and intracellular lipolysis in cancer. Cancer Lett.

[56] Yin H, Li W, Mo L, Deng S, Lin W, Ma C, Luo Z, Luo C, Hong H (2021). Adipose triglyceride lipase promotes the proliferation of colorectal cancer cells via enhancing the lipolytic pathway. J Cell Mol Med.

[57] Wang YY, Attane C, Milhas D, Dirat B, Dauvillier S, Guerard A, Gilhodes J, Lazar I, Alet N, Laurent V (2017). Mammary adipocytes stimulate breast cancer invasion through metabolic remodeling of tumor cells. JCI Insight.

[58] Iftikhar R, Penrose HM, King AN, Samudre JS, Collins ME, Hartono AB, Lee SB, Lau F, Baddoo M, Flemington EF (2021). Elevated ATGL in colon cancer cells and cancer stem cells promotes metabolic and tumorigenic reprogramming reinforced by obesity. Oncogenesis.

[59] Padanad MS, Konstantinidou G, Venkateswaran N, Melegari M, Rindhe S, Mitsche M, Yang C, Batten K, Huffman KE, Liu J (2016). Fatty acid oxidation mediated by Acyl-CoA synthetase long chain 3 is required for mutant kras lung tumorigenesis. Cell Rep.

[60] Wang L, Li C, Song Y, Yan Z (2020). Inhibition of carnitine palmitoyl transferase 1A-induced fatty acid oxidation suppresses cell progression in gastric cancer. Arch Biochem Biophys.

[61] Nimmakayala RK, Leon F, Rachagani S, Rauth S, Nallasamy P, Marimuthu S, Shailendra GK, Chhonker YS, Chugh S, Chirravuri R (2021). Metabolic programming of distinct cancer stem cells promotes metastasis of pancreatic ductal adenocarcinoma. Oncogene.

[62] Stockwell BR, Jiang X, Gu W (2020). Emerging mechanisms and disease relevance of ferroptosis. Trends Cell Biol.

[63] Dierge E, Debock E, Guilbaud C, Corbet C, Mignolet E, Mignard L, Bastien E, Dessy C, Larondelle Y, Feron O (2021). Peroxidation of n-3 and n-6 polyunsaturated fatty acids in the acidic tumor environment leads to ferroptosis-mediated anticancer effects. Cell Metab.

[64] Danielli M, Perne L, Jarc Jovicic E, Petan T (2023). Lipid droplets and polyunsaturated fatty acid trafficking: balancing life and death. Front Cell Dev Biol.

[65] Martin-Perez M, Urdiroz-Urricelqui U, Bigas C, Benitah SA (2022). The role of lipids in cancer progression and metastasis. Cell Metab.

[66] Liao P, Wang W, Wang W, Kryczek I, Li X, Bian Y, Sell A, Wei S, Grove S, Johnson JK (2022). CD8(+) T cells and fatty acids orchestrate tumor ferroptosis and immunity via ACSL4. Cancer Cell.

[67] Wang C, Shi M, Ji J, Cai Q, Zhao Q, Jiang J, Liu J, Zhang H, Zhu Z, Zhang J (2020). Stearoyl-CoA desaturase 1 (SCD1) facilitates the growth and anti-ferroptosis of gastric cancer cells and predicts poor prognosis of gastric cancer. Aging (Albany NY).

[68] Luo H, Wang X, Song S, Wang Y, Dan Q, Ge H (2022). Targeting stearoyl-coa desaturase enhances radiation induced ferroptosis and immunogenic cell death in esophageal squamous cell carcinoma. Oncoimmunology.

[69] Yi J, Zhu J, Wu J, Thompson CB, Jiang X (2020). Oncogenic activation of PI3K-AKT-mTOR signaling suppresses ferroptosis via SREBP-mediated lipogenesis. Proc Natl Acad Sci.

[70] Dong F, Mo Z, Eid W, Courtney KC, Zha X (2014). Akt inhibition promotes ABCA1-mediated cholesterol efflux to ApoA-I through suppressing mTORC1. PLoS ONE.

[71] Fruman DA, Chiu H, Hopkins BD, Bagrodia S, Cantley LC, Abraham RT (2017). The PI3K pathway in human disease. Cell.

[72] Calvisi DF, Wang C, Ho C, Ladu S, Lee SA, Mattu S, Destefanis G, Delogu S, Zimmermann A, Ericsson J (2011). Increased lipogenesis, induced by AKT-mTORC1-RPS6 signaling, promotes development of human hepatocellular carcinoma. Gastroenterology.

[73] Li L, Pilo GM, Li X, Cigliano A, Latte G, Che L, Joseph C, Mela M, Wang C, Jiang L (2016). Inactivation of fatty acid synthase impairs hepatocarcinogenesis driven by AKT in mice and humans. J Hepatol.

[74] Janku F, Yap TA, Meric-Bernstam F (2018). Targeting the PI3K pathway in cancer: are we making headway?. Nat Rev Clin Oncol.

[75] Yue S, Li J, Lee SY, Lee HJ, Shao T, Song B, Cheng L, Masterson TA, Liu X, Ratliff TL (2014). Cholesteryl ester accumulation induced by PTEN loss and PI3K/AKT activation underlies human prostate cancer aggressiveness. Cell Metab.

[76] Chen H, Qi Q, Wu N, Wang Y, Feng Q, Jin R, Jiang L (2022). Aspirin promotes RSL3-induced ferroptosis by suppressing mTOR/SREBP-1/SCD1-mediated lipogenesis in PIK3CA-mutatnt colorectal cancer. Redox Biol.

[77] Chen LL, Wang WJ (2021). p53 regulates lipid metabolism in cancer. Int J Biol Macromol.

[78] Liu J, Zhang C, Hu W, Feng Z (2019). Tumor suppressor p53 and metabolism. J Mol Cell Biol.

[79] Jiang P, Du W, Wang X, Mancuso A, Gao X, Wu M, Yang X (2011). p53 regulates biosynthesis through direct inactivation of glucose-6-phosphate dehydrogenase. Nat Cell Biol.

[80] Moon SH, Huang CH, Houlihan SL, Regunath K, Freed-Pastor WA, Morris JPT, Tschaharganeh DF, Kastenhuber ER, Barsotti AM, Culp-Hill R (2019). p53 represses the mevalonate pathway to mediate tumor suppression. Cell.

[81] Sanchez-Macedo N, Feng J, Faubert B, Chang N, Elia A, Rushing EJ, Tsuchihara K, Bungard D, Berger SL, Jones RG (2013). Depletion of the novel p53-target gene carnitine palmitoyltransferase 1C delays tumor growth in the neurofibromatosis type I tumor model. Cell Death Differ.

[82] Wahlstrom T, Henriksson MA (2015). Impact of MYC in regulation of tumor cell metabolism. Biochim Biophys Acta.

[83] Gouw AM, Margulis K, Liu NS, Raman SJ, Mancuso A, Toal GG, Tong L, Mosley A, Hsieh AL, Sullivan DK (2019). The MYC oncogene cooperates with sterol-regulated element-binding protein to regulate lipogenesis essential for neoplastic growth. Cell Metab.

[84] Chen J, Ding C, Chen Y, Hu W, Yu C, Peng C, Feng X, Cheng Q, Wu W, Lu Y (2021). ACSL4 reprograms fatty acid metabolism in hepatocellular carcinoma via c-Myc/SREBP1 pathway. Cancer Lett.

[85] Jia J, Che L, Cigliano A, Wang X, Peitta G, Tao J, Zhong S, Ribback S, Evert M, Chen X (2020). Pivotal role of fatty acid synthase in c-MYC Driven Hepatocarcinogenesis. Int J Mol Sci.

[86] Zhong C, Fan L, Yao F, Shi J, Fang W, Zhao H (2014). HMGCR is necessary for the tumorigenecity of esophageal squamous cell carcinoma and is regulated by Myc. Tumour Biol.

[87] Zhong C, Fan L, Li Z, Yao F, Zhao H (2019). SREBP2 is upregulated in esophageal squamous cell carcinoma and co-operates with c-Myc to regulate HMGCR expression. Mol Med Rep.

[88] Wise DR, DeBerardinis RJ, Mancuso A, Sayed N, Zhang XY, Pfeiffer HK, Nissim I, Daikhin E, Yudkoff M, McMahon SB (2008). Myc regulates a transcriptional program that stimulates mitochondrial glutaminolysis and leads to glutamine addiction. Proc Natl Acad Sci.

[89] Yoo HC, Yu YC, Sung Y, Han JM (2020). Glutamine reliance in cell metabolism. Exp Mol Med.

[90] Camarda R, Zhou AY, Kohnz RA, Balakrishnan S, Mahieu C, Anderton B, Eyob H, Kajimura S, Tward A, Krings G (2016). Inhibition of fatty acid oxidation as a therapy for MYC-overexpressing triple-negative breast cancer. Nat Med.

[91] Casciano JC, Perry C, Cohen-Nowak AJ, Miller KD, Vande Voorde J, Zhang Q, Chalmers S, Sandison ME, Liu Q, Hedley A (2020). MYC regulates fatty acid metabolism through a multigenic program in claudin-low triple negative breast cancer. Br J Cancer.

[92] Hinshaw DC, Shevde LA (2019). The tumor microenvironment innately modulates cancer progression. Cancer Res.

[93] Shao C, Yang F, Miao S, Liu W, Wang C, Shu Y, Shen H (2018). Role of hypoxia-induced exosomes in tumor biology. Mol Cancer.

[94] Munir R, Lisec J, Swinnen JV, Zaidi N (2019). Lipid metabolism in cancer cells under metabolic stress. Br J Cancer.

[95] Wise DR, Ward PS, Shay JE, Cross JR, Gruber JJ, Sachdeva UM, Platt JM, DeMatteo RG, Simon MC, Thompson CB (2011). Hypoxia promotes isocitrate dehydrogenase-dependent carboxylation of alpha-ketoglutarate to citrate to support cell growth and viability. Proc Natl Acad Sci.

[96] Metallo CM, Gameiro PA, Bell EL, Mattaini KR, Yang J, Hiller K, Jewell CM, Johnson ZR, Irvine DJ, Guarente L (2011). Reductive glutamine metabolism by IDH1 mediates lipogenesis under hypoxia. Nature.

[97] Liu Y, Ma Z, Zhao C, Wang Y, Wu G, Xiao J, McClain CJ, Li X, Feng W (2014). HIF-1alpha and HIF-2alpha are critically involved in hypoxia-induced lipid accumulation in hepatocytes through reducing PGC-1alpha-mediated fatty acid beta-oxidation. Toxicol Lett.

[98] Ezzeddini R, Taghikhani M, Salek Farrokhi A, Somi MH, Samadi N, Esfahani A, Rasaee MJ (2021). Downregulation of fatty acid oxidation by involvement of HIF-1alpha and PPARgamma in human gastric adenocarcinoma and related clinical significance. J Physiol Biochem.

[99] Du W, Zhang L, Brett-Morris A, Aguila B, Kerner J, Hoppel CL, Puchowicz M, Serra D, Herrero L, Rini BI (2017). HIF drives lipid deposition and cancer in ccRCC via repression of fatty acid metabolism. Nat Commun.

[100] Seo J, Jeong DW, Park JW, Lee KW, Fukuda J, Chun YS (2020). Fatty-acid-induced FABP5/HIF-1 reprograms lipid metabolism and enhances the proliferation of liver cancer cells. Commun Biol.

[101] Qiu B, Ackerman D, Sanchez DJ, Li B, Ochocki JD, Grazioli A, Bobrovnikova-Marjon E, Diehl JA, Keith B, Simon MC (2015). HIF2alpha-dependent lipid storage promotes endoplasmic reticulum homeostasis in clear-cell renal cell carcinoma. Cancer Discov.

[102] Kamphorst JJ, Cross JR, Fan J, de Stanchina E, Mathew R, White EP, Thompson CB, Rabinowitz JD (2013). Hypoxic and Ras-transformed cells support growth by scavenging unsaturated fatty acids from lysophospholipids. Proc Natl Acad Sci.

[103] Ackerman D, Tumanov S, Qiu B, Michalopoulou E, Spata M, Azzam A, Xie H, Simon MC, Kamphorst JJ (2018). Triglycerides promote lipid homeostasis during hypoxic stress by balancing fatty acid saturation. Cell Rep.

[104] Mylonis I, Simos G, Paraskeva E (2019). Hypoxia-inducible factors and the regulation of lipid metabolism. Cells.

[105] Boedtkjer E, Pedersen SF (2020). The acidic tumor microenvironment as a driver of cancer. Annu Rev Physiol.

[106] Maan M, Peters JM, Dutta M, Patterson AD (2018). Lipid metabolism and lipophagy in cancer. Biochem Biophys Res Commun.

[107] Corbet C, Bastien E, Santiago de Jesus JP, Dierge E, Martherus R, Vander Linden C, Doix B, Degavre C, Guilbaud C, Petit L (2020). TGFbeta2-induced formation of lipid droplets supports acidosis-driven EMT and the metastatic spreading of cancer cells. Nat Commun.

[108] Ding M, Zhang S, Guo Y, Yao J, Shen Q, Huang M, Chen W, Yu S, Zheng Y, Lin Y (2022). Tumor microenvironment acidity triggers lipid accumulation in liver cancer via scd1 activation. Mol Cancer Res.

[109] Kondo A, Yamamoto S, Nakaki R, Shimamura T, Hamakubo T, Sakai J, Kodama T, Yoshida T, Aburatani H, Osawa T (2017). Extracellular acidic pH activates the sterol regulatory element-binding protein 2 to promote tumor progression. Cell Rep.

[110] Vaupel P, Kallinowski F, Okunieff P (1989). Blood flow, oxygen and nutrient supply, and metabolic microenvironment of human tumors: a review. Cancer Res.

[111] Quail DF, Dannenberg AJ (2019). The obese adipose tissue microenvironment in cancer development and progression. Nat Rev Endocrinol.

[112] Wu Q, Li B, Li Z, Li J, Sun S, Sun S (2019). Cancer-associated adipocytes: key players in breast cancer progression. J Hematol Oncol.

[113] Yang D, Li Y, Xing L, Tan Y, Sun J, Zeng B, Xiang T, Tan J, Ren G, Wang Y (2018). Utilization of adipocyte-derived lipids and enhanced intracellular trafficking of fatty acids contribute to breast cancer progression. Cell Commun Signal.

[114] Attane C, Muller C (2020). Drilling for oil: tumor-surrounding adipocytes fueling cancer. Trends Cancer.

[115] Corn KC, Windham MA, Rafat M (2020). Lipids in the tumor microenvironment: from cancer progression to treatment. Prog Lipid Res.

[116] Tabuso M, Homer-Vanniasinkam S, Adya R, Arasaradnam RP (2017). Role of tissue microenvironment resident adipocytes in colon cancer. World J Gastroenterol.

[117] Balaban S, Shearer RF, Lee LS, van Geldermalsen M, Schreuder M, Shtein HC, Cairns R, Thomas KC, Fazakerley DJ, Grewal T (2017). Adipocyte lipolysis links obesity to breast cancer growth: adipocyte-derived fatty acids drive breast cancer cell proliferation and migration. Cancer Metab.

[118] Lien EC, Vander Heiden MG (2019). A framework for examining how diet impacts tumour metabolism. Nat Rev Cancer.

[119] Petrelli F, Cortellini A, Indini A, Tomasello G, Ghidini M, Nigro O, Salati M, Dottorini L, Iaculli A, Varricchio A (2021). Association of obesity with survival outcomes in patients with cancer: a systematic review and meta-analysis. JAMA Netw Open.

[120] Deng T, Lyon CJ, Bergin S, Caligiuri MA, Hsueh WA (2016). Obesity, inflammation, and cancer. Annu Rev Pathol.

[121] Beyaz S, Mana MD, Roper J, Kedrin D, Saadatpour A, Hong SJ, Bauer-Rowe KE, Xifaras ME, Akkad A, Arias E (2016). High-fat diet enhances stemness and tumorigenicity of intestinal progenitors. Nature.

[122] Kulkarni A, Bowers LW (2021). The role of immune dysfunction in obesity-associated cancer risk, progression, and metastasis. Cell Mol Life Sci.

[123] Kado T, Nawaz A, Takikawa A, Usui I, Tobe K (2019). Linkage of CD8(+) T cell exhaustion with high-fat diet-induced tumourigenesis. Sci Rep.

[124] Wang Z, Aguilar EG, Luna JI, Dunai C, Khuat LT, Le CT, Mirsoian A, Minnar CM, Stoffel KM, Sturgill IR (2019). Paradoxical effects of obesity on T cell function during tumor progression and PD-1 checkpoint blockade. Nat Med.

[125] Chen M, Zhang J, Sampieri K, Clohessy JG, Mendez L, Gonzalez-Billalabeitia E, Liu XS, Lee YR, Fung J, Katon JM (2018). An aberrant SREBP-dependent lipogenic program promotes metastatic prostate cancer. Nat Genet.

[126] Bousquenaud M, Fico F, Solinas G, Ruegg C, Santamaria-Martinez A (2018). Obesity promotes the expansion of metastasis-initiating cells in breast cancer. Breast Cancer Res.

[127] Pascual G, Dominguez D, Elosua-Bayes M, Beckedorff F, Laudanna C, Bigas C, Douillet D, Greco C, Symeonidi A, Hernandez I (2021). Dietary palmitic acid promotes a prometastatic memory via Schwann cells. Nature.

[128] Labbe DP, Zadra G, Yang M, Reyes JM, Lin CY, Cacciatore S, Ebot EM, Creech AL, Giunchi F, Fiorentino M (2019). High-fat diet fuels prostate cancer progression by rewiring the metabolome and amplifying the MYC program. Nat Commun.

[129] Li S, Wu T, Lu YX, Wang JX, Yu FH, Yang MZ, Huang YJ, Li ZJ, Wang SL, Huang L (2020). Obesity promotes gastric cancer metastasis via diacylglycerol acyltransferase 2-dependent lipid droplets accumulation and redox homeostasis. Redox Biol.

[130] Tutino V, De Nunzio V, Caruso MG, Veronese N, Lorusso D, Di Masi M, Benedetto ML, Notarnicola M (2019). Elevated AA/EPA ratio represents an inflammatory biomarker in tumor tissue of metastatic colorectal cancer patients. Int J Mol Sci.

[131] Matsuoka T, Adair JE, Lih FB, Hsi LC, Rubino M, Eling TE, Tomer KB, Yashiro M, Hirakawa K, Olden K (2010). Elevated dietary linoleic acid increases gastric carcinoma cell invasion and metastasis in mice. Br J Cancer.

[132] Chen X, Song E (2019). Turning foes to friends: targeting cancer-associated fibroblasts. Nat Rev Drug Discov.

[133] Kalluri R (2016). The biology and function of fibroblasts in cancer. Nat Rev Cancer.

[134] Sahai E, Astsaturov I, Cukierman E, DeNardo DG, Egeblad M, Evans RM, Fearon D, Greten FR, Hingorani SR, Hunter T (2020). A framework for advancing our understanding of cancer-associated fibroblasts. Nat Rev Cancer.

[135] Gong J, Lin Y, Zhang H, Liu C, Cheng Z, Yang X, Zhang J, Xiao Y, Sang N, Qian X (2020). Reprogramming of lipid metabolism in cancer-associated fibroblasts potentiates migration of colorectal cancer cells. Cell Death Dis.

[136] Zhang Y, Gu Z, Wan J, Lou X, Liu S, Wang Y, Bian Y, Wang F, Li Z, Qin Z (2022). Stearoyl-CoA Desaturase-1 dependent lipid droplets accumulation in cancer-associated fibroblasts facilitates the progression of lung cancer. Int J Biol Sci.

[137] Nardi F, Fitchev P, Franco OE, Ivanisevic J, Scheibler A, Hayward SW, Brendler CB, Welte MA, Crawford SE (2018). PEDF regulates plasticity of a novel lipid-MTOC axis in prostate cancer-associated fibroblasts. J Cell Sci.

[138] Hwang SH, Yang Y, Jung JH, Kim Y (2022). Oleic acid from cancer-associated fibroblast promotes cancer cell stemness by stearoyl-CoA desaturase under glucose-deficient condition. Cancer Cell Int.

[139] Peng S, Li Y, Huang M, Tang G, Xie Y, Chen D, Hu Y, Yu T, Cai J, Yuan Z (2022). Metabolomics reveals that CAF-derived lipids promote colorectal cancer peritoneal metastasis by enhancing membrane fluidity. Int J Biol Sci.

[140] Peng S, Chen D, Cai J, Yuan Z, Huang B, Li Y, Wang H, Luo Q, Kuang Y, Liang W (2021). Enhancing cancer-associated fibroblast fatty acid catabolism within a metabolically challenging tumor microenvironment drives colon cancer peritoneal metastasis. Mol Oncol.

[141] Li C, Teixeira AF, Zhu HJ, Ten Dijke P (2021). Cancer associated-fibroblast-derived exosomes in cancer progression. Mol Cancer.

[142] Li Z, Sun C, Qin Z (2021). Metabolic reprogramming of cancer-associated fibroblasts and its effect on cancer cell reprogramming. Theranostics.

[143] Radhakrishnan R, Ha JH, Jayaraman M, Liu J, Moxley KM, Isidoro C, Sood AK, Song YS, Dhanasekaran DN (2019). Ovarian cancer cell-derived lysophosphatidic acid induces glycolytic shift and cancer-associated fibroblast-phenotype in normal and peritumoral fibroblasts. Cancer Lett.

[144] Auciello FR, Bulusu V, Oon C, Tait-Mulder J, Berry M, Bhattacharyya S, Tumanov S, Allen-Petersen BL, Link J, Kendsersky ND (2019). A stromal lysolipid-autotaxin signaling axis promotes pancreatic tumor progression. Cancer Discov.

[145] Kock A, Larsson K, Bergqvist F, Eissler N, Elfman LHM, Raouf J, Korotkova M, Johnsen JI, Jakobsson PJ, Kogner P (2018). Inhibition of microsomal prostaglandin E synthase-1 in cancer-associated fibroblasts suppresses neuroblastoma tumor growth. EBioMedicine.

[146] Elwakeel E, Bruggemann M, Wagih J, Lityagina O, Elewa MAF, Han Y, Fromel T, Popp R, Nicolas AM, Schreiber Y (2022). Disruption of prostaglandin E2 signaling in cancer-associated fibroblasts limits mammary carcinoma growth but promotes metastasis. Cancer Res.

[147] Weigel C, Maczis MA, Palladino END, Green CD, Maceyka M, Guo C, Wang XY, Dozmorov MG, Milstien S, Spiegel S (2023). Sphingosine kinase 2 in stromal fibroblasts creates a hospitable tumor microenvironment in breast cancer. Cancer Res.

[148] Neuwirt H, Bouchal J, Kharaishvili G, Ploner C, Johrer K, Pitterl F, Weber A, Klocker H, Eder IE (2020). Cancer-associated fibroblasts promote prostate tumor growth and progression through upregulation of cholesterol and steroid biosynthesis. Cell Commun Signal.

[149] Zhang H, Deng T, Liu R, Ning T, Yang H, Liu D, Zhang Q, Lin D, Ge S, Bai M (2020). CAF secreted miR-522 suppresses ferroptosis and promotes acquired chemo-resistance in gastric cancer. Mol Cancer.

[150] Santi A, Caselli A, Ranaldi F, Paoli P, Mugnaioni C, Michelucci E, Cirri P (2015). Cancer associated fibroblasts transfer lipids and proteins to cancer cells through cargo vesicles supporting tumor growth. Biochim Biophys Acta.

[151] Lopes-Coelho F, Andre S, Felix A, Serpa J (2018). Breast cancer metabolic cross-talk: fibroblasts are hubs and breast cancer cells are gatherers of lipids. Mol Cell Endocrinol.

[152] Zhao H, Yang L, Baddour J, Achreja A, Bernard V, Moss T, Marini JC, Tudawe T, Seviour EG, San Lucas FA (2016). Tumor microenvironment derived exosomes pleiotropically modulate cancer cell metabolism. Elife.

[153] Zhu GQ, Tang Z, Huang R, Qu WF, Fang Y, Yang R, Tao CY, Gao J, Wu XL, Sun HX (2023). CD36(+) cancer-associated fibroblasts provide immunosuppressive microenvironment for hepatocellular carcinoma via secretion of macrophage migration inhibitory factor. Cell Discov.

[154] Chang CH, Qiu J, O'Sullivan D, Buck MD, Noguchi T, Curtis JD, Chen Q, Gindin M, Gubin MM, van der Windt GJ (2015). Metabolic competition in the tumor microenvironment is a driver of cancer progression. Cell.

[155] Luo Q, Zheng N, Jiang L, Wang T, Zhang P, Liu Y, Zheng P, Wang W, Xie G, Chen L (2020). Lipid accumulation in macrophages confers protumorigenic polarization and immunity in gastric cancer. Cancer Sci.

[156] Fridman WH, Pages F, Sautes-Fridman C, Galon J (2012). The immune contexture in human tumours: impact on clinical outcome. Nat Rev Cancer.

[157] Tumeh PC, Harview CL, Yearley JH, Shintaku IP, Taylor EJ, Robert L, Chmielowski B, Spasic M, Henry G, Ciobanu V (2014). PD-1 blockade induces responses by inhibiting adaptive immune resistance. Nature.

[158] Mellman I, Coukos G, Dranoff G (2011). Cancer immunotherapy comes of age. Nature.

[159] Pavlova NN, Thompson CB (2016). The emerging hallmarks of cancer metabolism. Cell Metab.

[160] DeBerardinis RJ, Chandel NS (2016). Fundamentals of cancer metabolism. Sci Adv.

[161] Ma X, Bi E, Lu Y, Su P, Huang C, Liu L, Wang Q, Yang M, Kalady MF, Qian J (2019). Cholesterol induces CD8(+) T cell exhaustion in the tumor microenvironment. Cell Metab.

[162] Zhang C, Yue C, Herrmann A, Song J, Egelston C, Wang T, Zhang Z, Li W, Lee H, Aftabizadeh M (2020). STAT3 activation-induced fatty acid oxidation in CD8(+) T effector cells is critical for obesity-promoted breast tumor growth. Cell Metab.

[163] Zhang Y, Kurupati R, Liu L, Zhou XY, Zhang G, Hudaihed A, Filisio F, Giles-Davis W, Xu X, Karakousis GC (2017). Enhancing CD8(+) T cell fatty acid catabolism within a metabolically challenging tumor microenvironment increases the efficacy of melanoma immunotherapy. Cancer Cell.

[164] Chowdhury PS, Chamoto K, Kumar A, Honjo T (2018). PPAR-induced fatty acid oxidation in t cells increases the number of tumor-reactive CD8(+) T Cells and facilitates anti-PD-1 therapy. Cancer Immunol Res.

[165] Xiao L, Ma X, Ye L, Su P, Xiong W, Bi E, Wang Q, Xian M, Yang M, Qian J (2022). IL-9/STAT3/fatty acid oxidation-mediated lipid peroxidation contributes to Tc9 cell longevity and enhanced antitumor activity. J Clin Invest.

[166] Patsoukis N, Bardhan K, Chatterjee P, Sari D, Liu B, Bell LN, Karoly ED, Freeman GJ, Petkova V, Seth P (2015). PD-1 alters T-cell metabolic reprogramming by inhibiting glycolysis and promoting lipolysis and fatty acid oxidation. Nat Commun.

[167] Ringel AE, Drijvers JM, Baker GJ, Catozzi A, Garcia-Canaveras JC, Gassaway BM, Miller BC, Juneja VR, Nguyen TH, Joshi S (2020). Obesity shapes metabolism in the tumor microenvironment to suppress anti-tumor immunity. Cell.

[168] Lin R, Zhang H, Yuan Y, He Q, Zhou J, Li S, Sun Y, Li DY, Qiu HB, Wang W (2020). Fatty acid oxidation controls CD8(+) tissue-resident memory T-cell survival in gastric adenocarcinoma. Cancer Immunol Res.

[169] Ma X, Xiao L, Liu L, Ye L, Su P, Bi E, Wang Q, Yang M, Qian J, Yi Q (2021). CD36-mediated ferroptosis dampens intratumoral CD8(+) T cell effector function and impairs their antitumor ability. Cell Metab.

[170] Manzo T, Prentice BM, Anderson KG, Raman A, Schalck A, Codreanu GS, Nava Lauson CB, Tiberti S, Raimondi A, Jones MA (2020). Accumulation of long-chain fatty acids in the tumor microenvironment drives dysfunction in intrapancreatic CD8+ T cells. J Exp Med.

[171] Ma X, Bi E, Huang C, Lu Y, Xue G, Guo X, Wang A, Yang M, Qian J, Dong C (2018). Cholesterol negatively regulates IL-9-producing CD8(+) T cell differentiation and antitumor activity. J Exp Med.

[172] Yang W, Bai Y, Xiong Y, Zhang J, Chen S, Zheng X, Meng X, Li L, Wang J, Xu C (2016). Potentiating the antitumour response of CD8(+) T cells by modulating cholesterol metabolism. Nature.

[173] Yan Y, Huang L, Liu Y, Yi M, Chu Q, Jiao D, Wu K (2022). Metabolic profiles of regulatory T cells and their adaptations to the tumor microenvironment: implications for antitumor immunity. J Hematol Oncol.

[174] Wang H, Franco F, Ho PC (2017). Metabolic regulation of tregs in cancer: opportunities for immunotherapy. Trends Cancer.

[175] Miska J, Lee-Chang C, Rashidi A, Muroski ME, Chang AL, Lopez-Rosas A, Zhang P, Panek WK, Cordero A, Han Y (2019). HIF-1alpha is a metabolic switch between glycolytic-driven migration and oxidative phosphorylation-driven immunosuppression of tregs in glioblastoma. Cell Rep.

[176] Lim SA, Wei J, Nguyen TM, Shi H, Su W, Palacios G, Dhungana Y, Chapman NM, Long L, Saravia J (2021). Lipid signalling enforces functional specialization of T(reg) cells in tumours. Nature.

[177] Kim MJ, Kim K, Park HJ, Kim GR, Hong KH, Oh JH, Son J, Park DJ, Kim D, Choi JM (2023). Deletion of PD-1 destabilizes the lineage identity and metabolic fitness of tumor-infiltrating regulatory T cells. Nat Immunol.

[178] Pacella I, Procaccini C, Focaccetti C, Miacci S, Timperi E, Faicchia D, Severa M, Rizzo F, Coccia EM, Bonacina F (2018). Fatty acid metabolism complements glycolysis in the selective regulatory T cell expansion during tumor growth. Proc Natl Acad Sci.

[179] Zeng H, Yang K, Cloer C, Neale G, Vogel P, Chi H (2013). mTORC1 couples immune signals and metabolic programming to establish T(reg)-cell function. Nature.

[180] Zhao R, Wan Q, Wang Y, Wu Y, Xiao S, Li Q, Shen X, Zhuang W, Zhou Y, Xia L (2020). M1-like TAMs are required for the efficacy of PD-L1/PD-1 blockades in gastric cancer. Oncoimmunology.

[181] Yang M, McKay D, Pollard JW, Lewis CE (2018). Diverse functions of macrophages in different tumor microenvironments. Cancer Res.

[182] Mehla K, Singh PK (2019). Metabolic regulation of macrophage polarization in cancer. Trends Cancer.

[183] Heusinkveld M, de Vos van Steenwijk PJ, Goedemans R, Ramwadhdoebe TH, Gorter A, Welters MJ, van Hall T, van der Burg SH (2011). M2 macrophages induced by prostaglandin E2 and IL-6 from cervical carcinoma are switched to activated M1 macrophages by CD4+ Th1 cells. J Immunol.

[184] Feng Y, Xiao M, Zhang Z, Cui R, Jiang X, Wang S, Bai H, Liu C, Zhang Z (2020). Potential interaction between lysophosphatidic acid and tumor-associated macrophages in ovarian carcinoma. J Inflamm (Lond).

[185] Linton SS, Abraham T, Liao J, Clawson GA, Butler PJ, Fox T, Kester M, Matters GL (2018). Tumor-promoting effects of pancreatic cancer cell exosomes on THP-1-derived macrophages. PLoS ONE.

[186] Flaherty SE, Grijalva A, Xu X, Ables E, Nomani A, Ferrante AW (2019). A lipase-independent pathway of lipid release and immune modulation by adipocytes. Science.

[187] Su P, Wang Q, Bi E, Ma X, Liu L, Yang M, Qian J, Yi Q (2020). Enhanced lipid accumulation and metabolism are required for the differentiation and activation of tumor-associated macrophages. Cancer Res.

[188] Liu S, Zhang H, Li Y, Zhang Y, Bian Y, Zeng Y, Yao X, Wan J, Chen X, Li J (2021). S100A4 enhances protumor macrophage polarization by control of PPAR-gamma-dependent induction of fatty acid oxidation. J Immunother Cancer.

[189] Yang P, Qin H, Li Y, Xiao A, Zheng E, Zeng H, Su C, Luo X, Lu Q, Liao M (2022). CD36-mediated metabolic crosstalk between tumor cells and macrophages affects liver metastasis. Nat Commun.

[190] Xu ZZ, Xu S, Kuhlmann A, Kaech SM (2020). The role of CD36 in macrophage lipid metabolism and function in tumor microenvironment. J Immunol.

[191] Rabold K, Aschenbrenner A, Thiele C, Boahen CK, Schiltmans A, Smit JWA, Schultze JL, Netea MG, Adema GJ, Netea-Maier RT (2020). Enhanced lipid biosynthesis in human tumor-induced macrophages contributes to their protumoral characteristics. J Immunother Cancer.

[192] Liu C, Chikina M, Deshpande R, Menk AV, Wang T, Tabib T, Brunazzi EA, Vignali KM, Sun M, Stolz DB (2019). Treg cells promote the SREBP1-dependent metabolic fitness of tumor-promoting macrophages via repression of CD8(+) T cell-derived interferon-gamma. Immunity.

[193] Bose D, Banerjee S, Chatterjee N, Das S, Saha M, Saha KD (2019). Inhibition of TGF-beta induced lipid droplets switches M2 macrophages to M1 phenotype. Toxicol In Vitro.

[194] Wu H, Han Y, Rodriguez Sillke Y, Deng H, Siddiqui S, Treese C, Schmidt F, Friedrich M, Keye J, Wan J (2019). Lipid droplet-dependent fatty acid metabolism controls the immune suppressive phenotype of tumor-associated macrophages. EMBO Mol Med.

[195] Zhang Q, Wang H, Mao C, Sun M, Dominah G, Chen L, Zhuang Z (2018). Fatty acid oxidation contributes to IL-1beta secretion in M2 macrophages and promotes macrophage-mediated tumor cell migration. Mol Immunol.

[196] Shu Y, Qin M, Song Y, Tang Q, Huang Y, Shen P, Lu Y (2020). M2 polarization of tumor-associated macrophages is dependent on integrin beta3 via peroxisome proliferator-activated receptor-gamma up-regulation in breast cancer. Immunology.

[197] Gordon SR, Maute RL, Dulken BW, Hutter G, George BM, McCracken MN, Gupta R, Tsai JM, Sinha R, Corey D (2017). PD-1 expression by tumour-associated macrophages inhibits phagocytosis and tumour immunity. Nature.

[198] Giovanelli P, Sandoval TA, Cubillos-Ruiz JR (2019). Dendritic cell metabolism and function in tumors. Trends Immunol.

[199] Herber DL, Cao W, Nefedova Y, Novitskiy SV, Nagaraj S, Tyurin VA, Corzo A, Cho HI, Celis E, Lennox B (2010). Lipid accumulation and dendritic cell dysfunction in cancer. Nat Med.

[200] O'Neill LA, Pearce EJ (2016). Immunometabolism governs dendritic cell and macrophage function. J Exp Med.

[201] Oh DS, Lee HK (2019). Autophagy protein ATG5 regulates CD36 expression and anti-tumor MHC class II antigen presentation in dendritic cells. Autophagy.

[202] Cubillos-Ruiz JR, Silberman PC, Rutkowski MR, Chopra S, Perales-Puchalt A, Song M, Zhang S, Bettigole SE, Gupta D, Holcomb K (2015). ER stress sensor XBP1 controls anti-tumor immunity by disrupting dendritic cell homeostasis. Cell.

[203] Zhao F, Xiao C, Evans KS, Theivanthiran T, DeVito N, Holtzhausen A, Liu J, Liu X, Boczkowski D, Nair S (2018). Paracrine Wnt5a-beta-catenin signaling triggers a metabolic program that drives dendritic cell tolerization. Immunity.

[204] Mace EM, Dongre P, Hsu HT, Sinha P, James AM, Mann SS, Forbes LR, Watkin LB, Orange JS (2014). Cell biological steps and checkpoints in accessing NK cell cytotoxicity. Immunol Cell Biol.

[205] Brand A, Singer K, Koehl GE, Kolitzus M, Schoenhammer G, Thiel A, Matos C, Bruss C, Klobuch S, Peter K (2016). LDHA-associated lactic acid production blunts tumor immunosurveillance by t and nk cells. Cell Metab.

[206] Harmon C, Robinson MW, Hand F, Almuaili D, Mentor K, Houlihan DD, Hoti E, Lynch L, Geoghegan J, O'Farrelly C (2019). Lactate-mediated acidification of tumor microenvironment induces apoptosis of liver-resident nk cells in colorectal liver metastasis. Cancer Immunol Res.

[207] Michelet X, Dyck L, Hogan A, Loftus RM, Duquette D, Wei K, Beyaz S, Tavakkoli A, Foley C, Donnelly R (2018). Metabolic reprogramming of natural killer cells in obesity limits antitumor responses. Nat Immunol.

[208] Gong Z, Li Q, Shi J, Liu ET, Shultz LD, Ren G (2022). Lipid-laden lung mesenchymal cells foster breast cancer metastasis via metabolic reprogramming of tumor cells and natural killer cells. Cell Metab.

[209] Kobayashi T, Lam PY, Jiang H, Bednarska K, Gloury R, Murigneux V, Tay J, Jacquelot N, Li R, Tuong ZK (2020). Increased lipid metabolism impairs NK cell function and mediates adaptation to the lymphoma environment. Blood.

[210] Qin WH, Yang ZS, Li M, Chen Y, Zhao XF, Qin YY, Song JQ, Wang BB, Yuan B, Cui XL (2020). High serum levels of cholesterol increase antitumor functions of nature killer cells and reduce growth of liver tumors in mice. Gastroenterology.

[211] Yan D, Adeshakin AO, Xu M, Afolabi LO, Zhang G, Chen YH, Wan X (2019). Lipid metabolic pathways confer the immunosuppressive function of myeloid-derived suppressor cells in tumor. Front Immunol.

[212] Marvel D, Gabrilovich DI (2015). Myeloid-derived suppressor cells in the tumor microenvironment: expect the unexpected. J Clin Invest.

[213] Xiang X, Poliakov A, Liu C, Liu Y, Deng ZB, Wang J, Cheng Z, Shah SV, Wang GJ, Zhang L (2009). Induction of myeloid-derived suppressor cells by tumor exosomes. Int J Cancer.

[214] Yan G, Zhao H, Zhang Q, Zhou Y, Wu L, Lei J, Wang X, Zhang J, Zhang X, Zheng L (2018). A RIPK3-PGE(2) circuit mediates myeloid-derived suppressor cell-potentiated colorectal carcinogenesis. Cancer Res.

[215] Porta C, Consonni FM, Morlacchi S, Sangaletti S, Bleve A, Totaro MG, Larghi P, Rimoldi M, Tripodo C, Strauss L (2020). Tumor-derived prostaglandin E2 promotes p50 NF-kappaB-dependent differentiation of monocytic MDSCs. Cancer Res.

[216] Mao Y, Sarhan D, Steven A, Seliger B, Kiessling R, Lundqvist A (2014). Inhibition of tumor-derived prostaglandin-e2 blocks the induction of myeloid-derived suppressor cells and recovers natural killer cell activity. Clin Cancer Res.

[217] Wang Y, Jia A, Bi Y, Wang Y, Liu G (2020). Metabolic regulation of myeloid-derived suppressor cell function in cancer. Cells.

[218] Al-Khami AA, Zheng L, Del Valle L, Hossain F, Wyczechowska D, Zabaleta J, Sanchez MD, Dean MJ, Rodriguez PC, Ochoa AC (2017). Exogenous lipid uptake induces metabolic and functional reprogramming of tumor-associated myeloid-derived suppressor cells. Oncoimmunology.

[219] Wu H, Weidinger C, Schmidt F, Keye J, Friedrich M, Yerinde C, Willimsky G, Qin Z, Siegmund B, Glauben R (2017). Oleate but not stearate induces the regulatory phenotype of myeloid suppressor cells. Sci Rep.

[220] Shi X, Pang S, Zhou J, Yan G, Sun J, Tan W (2022). Feedback loop between fatty acid transport protein 2 and receptor interacting protein 3 pathways promotes polymorphonuclear neutrophil myeloid-derived suppressor cells-potentiated suppressive immunity in bladder cancer. Mol Biol Rep.

[221] Adeshakin AO, Liu W, Adeshakin FO, Afolabi LO, Zhang M, Zhang G, Wang L, Li Z, Lin L, Cao Q (2021). Regulation of ROS in myeloid-derived suppressor cells through targeting fatty acid transport protein 2 enhanced anti-PD-L1 tumor immunotherapy. Cell Immunol.

[222] Hossain F, Al-Khami AA, Wyczechowska D, Hernandez C, Zheng L, Reiss K, Valle LD, Trillo-Tinoco J, Maj T, Zou W (2015). Inhibition of fatty acid oxidation modulates immunosuppressive functions of myeloid-derived suppressor cells and enhances cancer therapies. Cancer Immunol Res.

[223] Munir R, Lisec J, Swinnen JV, Zaidi N (2022). Too complex to fail? Targeting fatty acid metabolism for cancer therapy. Prog Lipid Res.

[224] Luo X, Cheng C, Tan Z, Li N, Tang M, Yang L, Cao Y (2017). Emerging roles of lipid metabolism in cancer metastasis. Mol Cancer.

[225] Yi M, Li J, Chen S, Cai J, Ban Y, Peng Q, Zhou Y, Zeng Z, Peng S, Li X (2018). Emerging role of lipid metabolism alterations in Cancer stem cells. J Exp Clin Cancer Res.

[226] Cao Y (2019). Adipocyte and lipid metabolism in cancer drug resistance. J Clin Invest.

[227] Goncalves AC, Richiardone E, Jorge J, Polonia B, Xavier CPR, Salaroglio IC, Riganti C, Vasconcelos MH, Corbet C, Sarmento-Ribeiro AB (2021). Impact of cancer metabolism on therapy resistance–clinical implications. Drug Resist Updat.

[228] Pascual G, Avgustinova A, Mejetta S, Martin M, Castellanos A, Attolini CS, Berenguer A, Prats N, Toll A, Hueto JA (2017). Targeting metastasis-initiating cells through the fatty acid receptor CD36. Nature.

[229] Watt MJ, Clark AK, Selth LA, Haynes VR, Lister N, Rebello R, Porter LH, Niranjan B, Whitby ST, Lo J (2019). Suppressing fatty acid uptake has therapeutic effects in preclinical models of prostate cancer. Sci Transl Med.

[230] Ligorio F, Di Cosimo S, Verderio P, Ciniselli CM, Pizzamiglio S, Castagnoli L, Dugo M, Galbardi B, Salgado R, Loi S (2022). Predictive role of CD36 expression in HER2-positive breast cancer patients receiving neoadjuvant trastuzumab. J Natl Cancer Inst.

[231] Feng WW, Wilkins O, Bang S, Ung M, Li J, An J, Del Genio C, Canfield K, DiRenzo J, Wells W (2019). CD36-Mediated metabolic rewiring of breast cancer cells promotes resistance to HER2-targeted therapies. Cell Rep.

[232] Farge T, Nakhle J, Lagarde D, Cognet G, Polley N, Castellano R, Nicolau ML, Bosc C, Sabatier M, Sahal A (2023). CD36 drives metastasis and relapse in acute myeloid leukemia. Cancer Res.

[233] Ye H, Adane B, Khan N, Sullivan T, Minhajuddin M, Gasparetto M, Stevens B, Pei S, Balys M, Ashton JM (2016). Leukemic stem cells evade chemotherapy by metabolic adaptation to an adipose tissue niche. Cell Stem Cell.

[234] Kubo M, Gotoh K, Eguchi H, Kobayashi S, Iwagami Y, Tomimaru Y, Akita H, Asaoka T, Noda T, Takeda Y (2020). Impact of CD36 on chemoresistance in pancreatic ductal adenocarcinoma. Ann Surg Oncol.

[235] O'Sullivan SE, Kaczocha M (2020). FABP5 as a novel molecular target in prostate cancer. Drug Discov Today.

[236] Carbonetti G, Converso C, Clement T, Wang C, Trotman LC, Ojima I, Kaczocha M (2020). Docetaxel/cabazitaxel and fatty acid binding protein 5 inhibitors produce synergistic inhibition of prostate cancer growth. Prostate.

[237] Mukherjee A, Chiang CY, Daifotis HA, Nieman KM, Fahrmann JF, Lastra RR, Romero IL, Fiehn O, Lengyel E (2020). Adipocyte-induced FABP4 expression in ovarian cancer cells promotes metastasis and mediates carboplatin resistance. Cancer Res.

[238] Alicea GM, Rebecca VW, Goldman AR, Fane ME, Douglass SM, Behera R, Webster MR, Kugel CH, Ecker BL, Caino MC (2020). Changes in aged fibroblast lipid metabolism induce age-dependent melanoma cell resistance to targeted therapy via the fatty acid transporter FATP2. Cancer Discov.

[239] Guo W, Ma J, Yang Y, Guo S, Zhang W, Zhao T, Yi X, Wang H, Wang S, Liu Y (2020). ATP-citrate lyase epigenetically potentiates oxidative phosphorylation to promote melanoma growth and adaptive resistance to MAPK inhibition. Clin Cancer Res.

[240] Wei X, Shi J, Lin Q, Ma X, Pang Y, Mao H, Li R, Lu W, Wang Y, Liu P (2021). Targeting ACLY attenuates tumor growth and acquired cisplatin resistance in ovarian cancer by inhibiting the PI3K-AKT pathway and activating the AMPK-ROS pathway. Front Oncol.

[241] Luo J, Hong Y, Lu Y, Qiu S, Chaganty BK, Zhang L, Wang X, Li Q, Fan Z (2017). Acetyl-CoA carboxylase rewires cancer metabolism to allow cancer cells to survive inhibition of the Warburg effect by cetuximab. Cancer Lett.

[242] Svensson RU, Parker SJ, Eichner LJ, Kolar MJ, Wallace M, Brun SN, Lombardo PS, Van Nostrand JL, Hutchins A, Vera L (2016). Inhibition of acetyl-CoA carboxylase suppresses fatty acid synthesis and tumor growth of non-small-cell lung cancer in preclinical models. Nat Med.

[243] Lally JSV, Ghoshal S, DePeralta DK, Moaven O, Wei L, Masia R, Erstad DJ, Fujiwara N, Leong V, Houde VP (2019). Inhibition of Acetyl-CoA carboxylase by phosphorylation or the inhibitor ND-654 suppresses lipogenesis and hepatocellular carcinoma. Cell Metab.

[244] Alkhouri N, Lawitz E, Noureddin M, DeFronzo R, Shulman GI (2020). GS-0976 (Firsocostat): an investigational liver-directed acetyl-CoA carboxylase (ACC) inhibitor for the treatment of non-alcoholic steatohepatitis (NASH). Expert Opin Investig Drugs.

[245] Li Y, Yang W, Zheng Y, Dai W, Ji J, Wu L, Cheng Z, Zhang J, Li J, Xu X (2023). Targeting fatty acid synthase modulates sensitivity of hepatocellular carcinoma to sorafenib via ferroptosis. J Exp Clin Cancer Res.

[246] Shueng PW, Chan HW, Lin WC, Kuo DY, Chuang HY (2022). Orlistat resensitizes sorafenib-resistance in hepatocellular carcinoma cells through modulating metabolism. Int J Mol Sci.

[247] Chuang HY, Lee YP, Lin WC, Lin YH, Hwang JJ (2019). Fatty acid inhibition sensitizes androgen-dependent and -independent prostate cancer to radiotherapy via FASN/NF-kappaB pathway. Sci Rep.

[248] Papaevangelou E, Almeida GS, Box C, deSouza NM, Chung YL (2018). The effect of FASN inhibition on the growth and metabolism of a cisplatin-resistant ovarian carcinoma model. Int J Cancer.

[249] Tadros S, Shukla SK, King RJ, Gunda V, Vernucci E, Abrego J, Chaika NV, Yu F, Lazenby AJ, Berim L (2017). De novo lipid synthesis facilitates gemcitabine resistance through endoplasmic reticulum stress in pancreatic cancer. Cancer Res.

[250] Ali A, Levantini E, Teo JT, Goggi J, Clohessy JG, Wu CS, Chen L, Yang H, Krishnan I, Kocher O (2018). Fatty acid synthase mediates EGFR palmitoylation in EGFR mutated non-small cell lung cancer. EMBO Mol Med.

[251] Wang H, Zhou Y, Xu H, Wang X, Zhang Y, Shang R, O'Farrell M, Roessler S, Sticht C, Stahl A (2022). Therapeutic efficacy of FASN inhibition in preclinical models of HCC. Hepatology.

[252] Liu Y, Gao GF, Minna JD, Williams NS, Westover KD (2021). Loss of wild type KRAS in KRAS(MUT) lung adenocarcinoma is associated with cancer mortality and confers sensitivity to FASN inhibitors. Lung Cancer.

[253] Sun P, Zhang X, Wang RJ, Ma QY, Xu L, Wang Y, Liao HP, Wang HL, Hu LD, Kong X (2021). PI3Kalpha inhibitor CYH33 triggers antitumor immunity in murine breast cancer by activating CD8(+)T cells and promoting fatty acid metabolism. J Immunother Cancer.

[254] Heuer TS, Ventura R, Mordec K, Lai J, Fridlib M, Buckley D, Kemble G (2017). FASN inhibition and taxane treatment combine to enhance anti-tumor efficacy in diverse xenograft tumor models through disruption of tubulin palmitoylation and microtubule organization and FASN inhibition-mediated effects on oncogenic signaling and gene expression. EBioMedicine.

[255] Alwarawrah Y, Hughes P, Loiselle D, Carlson DA, Darr DB, Jordan JL, Xiong J, Hunter LM, Dubois LG, Thompson JW (2016). Fasnall, a selective fasn inhibitor, shows potent anti-tumor activity in the MMTV-Neu model of HER2(+) breast cancer. Cell Chem Biol.

[256] Polonio-Alcala E, Porta R, Ruiz-Martinez S, Vasquez-Dongo C, Relat J, Bosch-Barrera J, Ciurana J, Puig T (2022). AZ12756122, a novel fatty acid synthase inhibitor, decreases resistance features in EGFR-TKI resistant EGFR-mutated NSCLC cell models. Biomed Pharmacother.

[257] Falchook G, Infante J, Arkenau HT, Patel MR, Dean E, Borazanci E, Brenner A, Cook N, Lopez J, Pant S (2021). First-in-human study of the safety, pharmacokinetics, and pharmacodynamics of first-in-class fatty acid synthase inhibitor TVB-2640 alone and with a taxane in advanced tumors. EClinicalMedicine.

[258] Kelly W, Diaz Duque AE, Michalek J, Konkel B, Caflisch L, Chen Y, Pathuri SC, Madhusudanannair Kunnuparampil V, Floyd J, Brenner A (2023). Phase II investigation of TVB-2640 (denifanstat) with bevacizumab in patients with first relapse high-grade astrocytoma. Clin Cancer Res.

[259] Tesfay L, Paul BT, Konstorum A, Deng Z, Cox AO, Lee J, Furdui CM, Hegde P, Torti FM, Torti SV (2019). Stearoyl-CoA desaturase 1 protects ovarian cancer cells from ferroptotic cell death. Cancer Res.

[260] Pisanu ME, Maugeri-Sacca M, Fattore L, Bruschini S, De Vitis C, Tabbi E, Bellei B, Migliano E, Kovacs D, Camera E (2018). Inhibition of Stearoyl-CoA desaturase 1 reverts BRAF and MEK inhibition-induced selection of cancer stem cells in BRAF-mutated melanoma. J Exp Clin Cancer Res.

[261] von Roemeling CA, Marlow LA, Wei JJ, Cooper SJ, Caulfield TR, Wu K, Tan WW, Tun HW, Copland JA (2013). Stearoyl-CoA desaturase 1 is a novel molecular therapeutic target for clear cell renal cell carcinoma. Clin Cancer Res.

[262] Dai S, Yan Y, Xu Z, Zeng S, Qian L, Huo L, Li X, Sun L, Gong Z (2017). SCD1 confers temozolomide resistance to human glioma cells via the Akt/GSK3beta/beta-catenin signaling axis. Front Pharmacol.

[263] Ma MKF, Lau EYT, Leung DHW, Lo J, Ho NPY, Cheng LKW, Ma S, Lin CH, Copland JA, Ding J (2017). Stearoyl-CoA desaturase regulates sorafenib resistance via modulation of ER stress-induced differentiation. J Hepatol.

[264] Huang Q, Wang Q, Li D, Wei X, Jia Y, Zhang Z, Ai B, Cao X, Guo T, Liao Y (2019). Co-administration of 20(S)-protopanaxatriol (g-PPT) and EGFR-TKI overcomes EGFR-TKI resistance by decreasing SCD1 induced lipid accumulation in non-small cell lung cancer. J Exp Clin Cancer Res.

[265] Pisanu ME, Noto A, De Vitis C, Morrone S, Scognamiglio G, Botti G, Venuta F, Diso D, Jakopin Z, Padula F (2017). Blockade of Stearoyl-CoA-desaturase 1 activity reverts resistance to cisplatin in lung cancer stem cells. Cancer Lett.

[266] Talebi A, Dehairs J, Rambow F, Rogiers A, Nittner D, Derua R, Vanderhoydonc F, Duarte JAG, Bosisio F, Van den Eynde K (2018). Sustained SREBP-1-dependent lipogenesis as a key mediator of resistance to BRAF-targeted therapy. Nat Commun.

[267] Yin F, Feng F, Wang L, Wang X, Li Z, Cao Y (2019). SREBP-1 inhibitor Betulin enhances the antitumor effect of Sorafenib on hepatocellular carcinoma via restricting cellular glycolytic activity. Cell Death Dis.

[268] Dechaphunkul T, Arundon T, Raungkhajon P, Jiratrachu R, Geater SL, Dechaphunkul A (2022). Benefits of immunonutrition in patients with head and neck cancer receiving chemoradiation: a phase II randomized, double-blind study. Clin Nutr.

[269] Mullen PJ, Yu R, Longo J, Archer MC, Penn LZ (2016). The interplay between cell signalling and the mevalonate pathway in cancer. Nat Rev Cancer.

[270] Hagiwara N, Watanabe M, Iizuka-Ohashi M, Yokota I, Toriyama S, Sukeno M, Tomosugi M, Sowa Y, Hongo F, Mikami K (2018). Mevalonate pathway blockage enhances the efficacy of mTOR inhibitors with the activation of retinoblastoma protein in renal cell carcinoma. Cancer Lett.

[271] Kim JS, Turbov J, Rosales R, Thaete LG, Rodriguez GC (2019). Combination simvastatin and metformin synergistically inhibits endometrial cancer cell growth. Gynecol Oncol.

[272] Shojaei S, Koleini N, Samiei E, Aghaei M, Cole LK, Alizadeh J, Islam MI, Vosoughi AR, Albokashy M, Butterfield Y (2020). Simvastatin increases temozolomide-induced cell death by targeting the fusion of autophagosomes and lysosomes. FEBS J.

[273] Zhang Y, Liu Y, Duan J, Wang H, Zhang Y, Qiao K, Wang J (2019). Cholesterol depletion sensitizes gallbladder cancer to cisplatin by impairing DNA damage response. Cell Cycle.

[274] Peng Y, He G, Tang D, Xiong L, Wen Y, Miao X, Hong Z, Yao H, Chen C, Yan S (2017). Lovastatin inhibits cancer stem cells and sensitizes to chemo-and photodynamic therapy in nasopharyngeal carcinoma. J Cancer.

[275] Han JY, Lee SH, Yoo NJ, Hyung LS, Moon YJ, Yun T, Kim HT, Lee JS (2011). A randomized phase II study of gefitinib plus simvastatin versus gefitinib alone in previously treated patients with advanced non-small cell lung cancer. Clin Cancer Res.

[276] Yulian ED, Siregar NC, Bajuadji (2021). Combination of simvastatin and FAC improves response to neoadjuvant chemotherapy in locally advanced breast cancer. Cancer Res Treat.

[277] Kim ST, Kang JH, Lee J, Park SH, Park JO, Park YS, Lim HY, Hwang IG, Lee SC, Park KW (2014). Simvastatin plus capecitabine-cisplatin versus placebo plus capecitabine-cisplatin in patients with previously untreated advanced gastric cancer: a double-blind randomised phase 3 study. Eur J Cancer.

[278] Siltari A, Riikonen J, Koskimaki J, Pakarainen T, Ettala O, Bostrom P, Seikkula H, Kotsar A, Tammela T, Helminen M (2022). Randomised double-blind phase 3 clinical study testing impact of atorvastatin on prostate cancer progression after initiation of androgen deprivation therapy: study protocol. BMJ Open.

[279] de la Cueva A, Ramirez de Molina A, Alvarez-Ayerza N, Ramos MA, Cebrian A, Del Pulgar TG, Lacal JC (2013). Combined 5-FU and ChoKalpha inhibitors as a new alternative therapy of colorectal cancer: evidence in human tumor-derived cell lines and mouse xenografts. PLoS ONE.

[280] Mazarico JM, Sanchez-Arevalo Lobo VJ, Favicchio R, Greenhalf W, Costello E, Carrillo-de Santa Pau E, Marques M, Lacal JC, Aboagye E, Real FX (2016). Choline kinase alpha (CHKalpha) as a therapeutic target in pancreatic ductal adenocarcinoma: expression, predictive value, and sensitivity to inhibitors. Mol Cancer Ther.

[281] Abalsamo L, Spadaro F, Bozzuto G, Paris L, Cecchetti S, Lugini L, Iorio E, Molinari A, Ramoni C, Podo F (2012). Inhibition of phosphatidylcholine-specific phospholipase C results in loss of mesenchymal traits in metastatic breast cancer cells. Breast Cancer Res.

[282] Chen Q, Hongu T, Sato T, Zhang Y, Ali W, Cavallo JA, van der Velden A, Tian H, Di Paolo G, Nieswandt B (2012). Key roles for the lipid signaling enzyme phospholipase d1 in the tumor microenvironment during tumor angiogenesis and metastasis. Sci Signal.

[283] Wang D, Cabalag CS, Clemons NJ, DuBois RN (2021). Cyclooxygenases and prostaglandins in tumor immunology and microenvironment of gastrointestinal cancer. Gastroenterology.

[284] Samudio I, Harmancey R, Fiegl M, Kantarjian H, Konopleva M, Korchin B, Kaluarachchi K, Bornmann W, Duvvuri S, Taegtmeyer H (2010). Pharmacologic inhibition of fatty acid oxidation sensitizes human leukemia cells to apoptosis induction. J Clin Invest.

[285] Yu S, Li Q, Yu Y, Cui Y, Li W, Liu T, Liu F (2020). Activated HIF1alpha of tumor cells promotes chemoresistance development via recruiting GDF15-producing tumor-associated macrophages in gastric cancer. Cancer Immunol Immunother.

[286] Tan Z, Xiao L, Tang M, Bai F, Li J, Li L, Shi F, Li N, Li Y, Du Q (2018). Targeting CPT1A-mediated fatty acid oxidation sensitizes nasopharyngeal carcinoma to radiation therapy. Theranostics.

[287] Iwamoto H, Abe M, Yang Y, Cui D, Seki T, Nakamura M, Hosaka K, Lim S, Wu J, He X (2018). Cancer lipid metabolism confers antiangiogenic drug resistance. Cell Metab.

[288] Holubarsch CJ, Rohrbach M, Karrasch M, Boehm E, Polonski L, Ponikowski P, Rhein S (2007). A double-blind randomized multicentre clinical trial to evaluate the efficacy and safety of two doses of etomoxir in comparison with placebo in patients with moderate congestive heart failure: the ERGO (etomoxir for the recovery of glucose oxidation) study. Clin Sci (Lond).

[289] Reyes-Castellanos G, Abdel Hadi N, Gallardo-Arriaga S, Masoud R, Garcia J, Lac S, El Kaoutari A, Gicquel T, Planque M, Fendt SM (2023). Combining the antianginal drug perhexiline with chemotherapy induces complete pancreatic cancer regression in vivo. Iscience.

[290] Zhu J, Wu G, Song L, Cao L, Tan Z, Tang M, Li Z, Shi D, Zhang S, Li J (2019). NKX2-8 deletion-induced reprogramming of fatty acid metabolism confers chemoresistance in epithelial ovarian cancer. EBioMedicine.

[291] Wang T, Fahrmann JF, Lee H, Li YJ, Tripathi SC, Yue C, Zhang C, Lifshitz V, Song J, Yuan Y (2018). JAK/STAT3-regulated fatty acid beta-oxidation is critical for breast cancer stem cell self-renewal and chemoresistance. Cell Metab.

[292] Ricciardi MR, Mirabilii S, Allegretti M, Licchetta R, Calarco A, Torrisi MR, Foa R, Nicolai R, Peluso G, Tafuri A (2015). Targeting the leukemia cell metabolism by the CPT1a inhibition: functional preclinical effects in leukemias. Blood.

[293] Wu C, Dai C, Li X, Sun M, Chu H, Xuan Q, Yin Y, Fang C, Yang F, Jiang Z (2022). AKR1C3-dependent lipid droplet formation confers hepatocellular carcinoma cell adaptability to targeted therapy. Theranostics.

[294] Chen L, Ma WL, Cheng WC, Yang JC, Wang HC, Su YT, Ahmad A, Hung YC, Chang WC (2020). Targeting lipid droplet lysophosphatidylcholine for cisplatin chemotherapy. J Cell Mol Med.

[295] Cotte AK, Aires V, Fredon M, Limagne E, Derangere V, Thibaudin M, Humblin E, Scagliarini A, de Barros JP, Hillon P (2018). Lysophosphatidylcholine acyltransferase 2-mediated lipid droplet production supports colorectal cancer chemoresistance. Nat Commun.

[296] Tirinato L, Pagliari F, Di Franco S, Sogne E, Marafioti MG, Jansen J, Falqui A, Todaro M, Candeloro P, Liberale C (2020). ROS and Lipid Droplet accumulation induced by high glucose exposure in healthy colon and Colorectal Cancer Stem Cells. Genes Dis.

[297] Senkal CE, Salama MF, Snider AJ, Allopenna JJ, Rana NA, Koller A, Hannun YA, Obeid LM (2017). Ceramide is metabolized to acylceramide and stored in lipid droplets. Cell Metab.

[298] Hernandez-Corbacho MJ, Obeid LM (2019). A novel role for DGATs in cancer. Adv Biol Regul.

[299] Nistico C, Pagliari F, Chiarella E, Fernandes Guerreiro J, Marafioti MG, Aversa I, Genard G, Hanley R, Garcia-Calderon D, Bond HM (2021). Lipid droplet biosynthesis impairment through DGAT2 inhibition sensitizes MCF7 breast cancer cells to radiation. Int J Mol Sci.

[300] Li J, Gu D, Lee SS, Song B, Bandyopadhyay S, Chen S, Konieczny SF, Ratliff TL, Liu X, Xie J (2016). Abrogating cholesterol esterification suppresses growth and metastasis of pancreatic cancer. Oncogene.

[301] Jiang Y, Sun A, Zhao Y, Ying W, Sun H, Yang X, Xing B, Sun W, Ren L, Hu B (2019). Proteomics identifies new therapeutic targets of early-stage hepatocellular carcinoma. Nature.

[302] Lee SS, Li J, Tai JN, Ratliff TL, Park K, Cheng JX (2015). Avasimibe encapsulated in human serum albumin blocks cholesterol esterification for selective cancer treatment. ACS Nano.

[303] Li M, Yang Y, Wei J, Cun X, Lu Z, Qiu Y, Zhang Z, He Q (2018). Enhanced chemo-immunotherapy against melanoma by inhibition of cholesterol esterification in CD8(+) T cells. Nanomedicine.

[304] Li J, Qu X, Tian J, Zhang JT, Cheng JX (2018). Cholesterol esterification inhibition and gemcitabine synergistically suppress pancreatic ductal adenocarcinoma proliferation. PLoS ONE.

[305] Ueno G, Iwagami Y, Kobayashi S, Mitsufuji S, Yamada D, Tomimaru Y, Akita H, Asaoka T, Noda T, Gotoh K (2022). ACAT-1-regulated cholesteryl ester accumulation modulates gemcitabine resistance in biliary tract cancer. Ann Surg Oncol.

[306] June CH, Riddell SR, Schumacher TN (2015). Adoptive cellular therapy: a race to the finish line. Sci Transl Med.

[307] Sanmamed MF, Chen L (2018). A paradigm shift in cancer immunotherapy: from enhancement to normalization. Cell.

[308] Chowdhury PS, Chamoto K, Honjo T (2018). Combination therapy strategies for improving PD-1 blockade efficacy: a new era in cancer immunotherapy. J Intern Med.

[309] Yang Y, Hsu JM, Sun L, Chan LC, Li CW, Hsu JL, Wei Y, Xia W, Hou J, Qiu Y (2019). Palmitoylation stabilizes PD-L1 to promote breast tumor growth. Cell Res.

[310] Yao H, Lan J, Li C, Shi H, Brosseau JP, Wang H, Lu H, Fang C, Zhang Y, Liang L (2019). Inhibiting PD-L1 palmitoylation enhances T-cell immune responses against tumours. Nat Biomed Eng.

[311] Cantini L, Pecci F, Hurkmans DP, Belderbos RA, Lanese A, Copparoni C, Aerts S, Cornelissen R, Dumoulin DW, Fiordoliva I (2021). High-intensity statins are associated with improved clinical activity of PD-1 inhibitors in malignant pleural mesothelioma and advanced non-small cell lung cancer patients. Eur J Cancer.

[312] Jiang N, Xie B, Xiao W, Fan M, Xu S, Duan Y, Hamsafar Y, Evans AC, Huang J, Zhou W (2022). Fatty acid oxidation fuels glioblastoma radioresistance with CD47-mediated immune evasion. Nat Commun.

[313] Wan H, Xu B, Zhu N, Ren B (2020). PGC-1alpha activator-induced fatty acid oxidation in tumor-infiltrating CTLs enhances effects of PD-1 blockade therapy in lung cancer. Tumori.

[314] Saibil SD, St Paul M, Laister RC, Garcia-Batres CR, Israni-Winger K, Elford AR, Grimshaw N, Robert-Tissot C, Roy DG, Jones RG (2019). Activation of peroxisome proliferator-activated receptors alpha and delta synergizes with inflammatory signals to enhance adoptive cell therapy. Cancer Res.

[315] Kansal V, Burnham AJ, Kinney BLC, Saba NF, Paulos C, Lesinski GB, Buchwald ZS, Schmitt NC (2023). Statin drugs enhance responses to immune checkpoint blockade in head and neck cancer models. J Immunother Cancer.

[316] Takada K, Shimokawa M, Takamori S, Shimamatsu S, Hirai F, Tagawa T, Okamoto T, Hamatake M, Tsuchiya-Kawano Y, Otsubo K (2022). A propensity score-matched analysis of the impact of statin therapy on the outcomes of patients with non-small-cell lung cancer receiving anti-PD-1 monotherapy: a multicenter retrospective study. BMC Cancer.

[317] Liu X, Bao X, Hu M, Chang H, Jiao M, Cheng J, Xie L, Huang Q, Li F, Li CY (2020). Inhibition of PCSK9 potentiates immune checkpoint therapy for cancer. Nature.

[318] Schmidt NM, Wing PAC, Diniz MO, Pallett LJ, Swadling L, Harris JM, Burton AR, Jeffery-Smith A, Zakeri N, Amin OE (2021). Targeting human Acyl-CoA: cholesterol acyltransferase as a dual viral and T cell metabolic checkpoint. Nat Commun.

[319] Giro-Perafita A, Palomeras S, Lum DH, Blancafort A, Vinas G, Oliveras G, Perez-Bueno F, Sarrats A, Welm AL, Puig T (2016). Preclinical evaluation of fatty acid synthase and EGFR inhibition in triple-negative breast cancer. Clin Cancer Res.

[320] Li CF, Fang FM, Chen YY, Liu TT, Chan TC, Yu SC, Chen LT, Huang HY (2017). Overexpressed fatty acid synthase in gastrointestinal stromal tumors: targeting a progression-associated metabolic driver enhances the antitumor effect of imatinib. Clin Cancer Res.

[321] Li X, Wu JB, Chung LW, Huang WC (2015). Anti-cancer efficacy of SREBP inhibitor, alone or in combination with docetaxel, in prostate cancer harboring p53 mutations. Oncotarget.

[322] Shim JK, Choi S, Yoon SJ, Choi RJ, Park J, Lee EH, Cho HJ, Lee S, Teo WY, Moon JH (2022). Etomoxir, a carnitine palmitoyltransferase 1 inhibitor, combined with temozolomide reduces stemness and invasiveness in patient-derived glioblastoma tumorspheres. Cancer Cell Int.

[323] Han S, Wei R, Zhang X, Jiang N, Fan M, Huang JH, Xie B, Zhang L, Miao W, Butler AC (2019). CPT1A/2-mediated FAO enhancement-a metabolic target in radioresistant breast cancer. Front Oncol.

[324] Mao S, Ling Q, Pan J, Li F, Huang S, Ye W, Wei W, Lin X, Qian Y, Wang Y (2021). Inhibition of CPT1a as a prognostic marker can synergistically enhance the antileukemic activity of ABT199. J Transl Med.

[325] Bandyopadhyay S, Li J, Traer E, Tyner JW, Zhou A, Oh ST, Cheng JX (2017). Cholesterol esterification inhibition and imatinib treatment synergistically inhibit growth of BCR-ABL mutation-independent resistant chronic myelogenous leukemia. PLoS ONE.

[326] Lei J, Wang H, Zhu D, Wan Y, Yin L (2020). Combined effects of avasimibe immunotherapy, doxorubicin chemotherapy, and metal-organic frameworks nanoparticles on breast cancer. J Cell Physiol.

